# Gamified Assessment of Cognitive Impulsivity in Eating Disorders and Mental Ill-Health: Mixed Methods Study Incorporating Lived Experience Co-Design and Evaluation

**DOI:** 10.2196/79784

**Published:** 2026-06-03

**Authors:** Emily Colton, Courtney McLean, Alexandra Anderson, Lauren Hanegraaf, Antonio Verdejo-Garcia

**Affiliations:** 1 School of Psychological Sciences, Monash University, Clayton, Australia; 2 Eastern Health Clinical School, Monash University, 110 Church Street, Richmond, Victoria, 3121, Australia, 61 384138668

**Keywords:** eating disorders, cognitive impulsivity, cognitive assessment, lived experience co-design, user-centred design, gamification, thematic analysis

## Abstract

**Background:**

Cognitive impulsivity is a multifaceted construct associated with symptom severity, functional impairment, and poor quality of life in eating disorders (EDs) and mental ill-health. However, objective assessment of cognitive impulsivity is piecemeal and complex, with many assessment tools lacking psychometric evaluation and ecological validity. Furthermore, validated assessment tools are rarely perceived to be engaging or meaningful by individuals who complete them, limiting their utility in research and forming a barrier to clinical translation. Thus, although cognitive impulsivity predicts treatment engagement and outcomes, it is rarely assessed or addressed in a clinical context.

**Objective:**

We aimed to redesign and evaluate the Cognitive Impulsivity Suite (CIS), a validated gamified assessment battery of cognitive impulsivity, through user-centered co-design, agile game development, and user-centered evaluation. This collaborative study partnered researchers with individuals with lived experiences (LEs) of EDs and commonly co-occurring mental ill-health, and game development experts.

**Methods:**

In a sequential mixed methods design, we first defined user requirements through inductive thematic analysis of data from 2 focus groups incorporating 7 individuals with LE of EDs and commonly co-occurring mental health conditions (mean age 27.6, SD 7.03 y; 4 women, 2 men, 1 nonbinary), researchers, clinicians, and technology professionals. Agile game development was achieved through 6-week-long sprints, involving game developers and a play-testing team of researchers. During LE evaluation, we collected and analyzed data from an expanded sample (n=18; mean age 30.5, SD 6.56 y; 12 women, 3 men, 2 nonbinary), using a pragmatic blending of qualitative and quantitative research methods. This included inductive and deductive thematic analysis of “thinking aloud” data, descriptive statistics, and ANOVA tests of the Game User Experience Satisfaction Scale, short version (GUESS-18) surveys.

**Results:**

We co-designed guiding principles and ideas for aesthetics, story concepts, and gameplay features, which closely aligned with leading theories of psychological well-being, clinical evidence concerning ED recovery, and gamification frameworks. Qualitative evaluations of the new tool, CIS *Papillon Park*, showed user engagement and motivation were driven by opportunities for autonomy, personal accomplishment, and simulated interpersonal relationships, aligning with approaches to gamification based on self-determination theory. In quantitative evaluation, a mean GUESS-18 composite score of 45.9 (SD 9.85) showed CIS *Papillon Park* achieved sound overall user satisfaction, with subscale scores revealing strengths in usability, narrative, visual and audio aesthetics, and personal gratification.

**Conclusions:**

The contributions of this study are grounded in its integration of user-centered co-design and evaluation, agile game development, and theory-driven approaches to cognitive assessment and gamification, to redesign and evaluate a validated online task battery. The resulting CIS *Papillon Park* combines appealing aesthetics, gamification elements that address cognitive, emotional, and social needs, and accessible playing experiences, which maximized user satisfaction and engagement while prioritizing psychological safety. Next steps involve psychometric evaluation and dissemination.

## Introduction

Cognitive impulsivity is a multifaceted construct that describes rapid, unplanned reactions without sufficient regard for their potential consequences, which arise from alterations in multiple processes, including attentional control, reflection, and feedback monitoring or shifting [1-3]. Distinctive patterns of cognitive impulsivity are well-documented characteristics of eating disorders (EDs) [4-8]. Furthermore, similar patterns are observed in conditions that commonly co-occur with EDs, such as mood and anxiety disorders, obsessive-compulsive disorder, substance and behavioral addictions, and attention-deficit/hyperactivity disorder [9-14]. Although causal pathways remain uncertain, elevated cognitive impulsivity has been shown to predict future onset of these disorders [15], to become more pronounced with illness duration [16], and to predict treatment outcomes [17]. Furthermore, research indicates greater severity of cognitive dysfunction in EDs is associated with symptom severity, functional impairment, and poorer quality of life [18,19]. Importantly, objective assessment of cognitive impulsivity has improved our understanding of shared and unique risk, maintenance, and treatment response mechanisms across EDs and commonly co-occurring conditions [20-24]. Nomothetic assessment of cognitive impulsivity has also facilitated the identification of novel treatment targets and neurocognitive interventions, while idiographic assessment may help identify which individuals will most benefit from those treatments [25-30]. As such, incorporating objective assessment of cognitive functioning in clinical assessment, diagnosis, and treatment planning may offer an opportunity to bridge critical treatment gaps, and help us to better understand and respond to complex clinical phenomena, such as diagnostic crossover, heterogeneity, and comorbidity [31-33]. However, despite persistent calls for this translation, objective assessment of cognitive impulsivity remains absent from routine clinical practice [34,35].

Specific barriers have been identified that limit the research impact and translation of cognitive impulsivity assessments. The first concern is the quality and quantity of existing tools, whereby many measure discrete facets of cognitive impulsivity and fail to predict real-world impulsive behaviors [36], and lack robust psychometric or normative evaluation [24,37-41]. Furthermore, the sheer array of tools, paradigms, and analysis methods creates onerous administration for clinicians and researchers, and limits replicability, comprehensiveness, and comparison [42,43]. Finally, a few of the evidence-based tools are well-accepted or engaging to users, leading to performance invalidity [44-46], limiting research participation and uptake in clinical or self-assessment contexts [47-53]. One approach to address these barriers is gamification—the process of creating opportunities for subjective value [54] through the introduction of game-like elements and mechanics [55]. Central to this approach is that gamification activates intrinsic motivational processes, thus drawing on self-determination theory, which posits human behavior is motivated by needs for competence, autonomy, and relatedness [56]. Gamification also reflects principles from other influential theories and frameworks, including social learning theory, social cognitive theory, behaviorism, and flow [57]. Gamification elements may therefore target a range of mechanisms to strengthen intrinsic motivation—cognitive (eg, achievement, progression, and mastery), emotional (eg, enjoyment, satisfaction, and immersion), or social (eg, collaboration and teamwork) [58,59]. Evidence suggests these can be achieved through various “gameful affordances” [54], for example, decomposing demanding activities into clear, achievable subtasks; creating meaning through narrative, characters, and storytelling; providing feedback, rewards, and visible indicators of progress; enabling exploration and customization; including teammates and social supports; and calibrating difficulty to optimal levels for each player [58-61]. Together with game design elements, such as aesthetic appeal, these in turn foster the desired player behavior or outcome, such as long-term user engagement, satisfaction, and consistent effort [57,62-66]. The Cognitive Impulsivity Suite (CIS) is an online task battery that was designed using these principles [3]. The CIS comprehensively assesses key cognitive processes associated with impulsive behaviors in an integrated environment, featuring a cohesive “Wild West” story and visual design. Multiple mechanisms are assessed in a single session, allowing outcomes to be analyzed and interpreted in a holistic manner, while using gamification to maximize consumer engagement [67-69]. Research has demonstrated this integrated task structure to be valid and reliable in assessing cognitive impulsivity in clinical and community samples, whether administered online or in-person [3,70,71]. However, it has also highlighted opportunities to broaden the reach and enhance the impact of the tool, particularly by advancing its clinical translation, and to improve user satisfaction, including for those with lived experience (LE) of EDs and commonly co-occurring conditions [72,73].

Lack of input from users is a second well-documented barrier limiting the research impact and clinical translation of cognitive assessments and similar technology-enabled tools [74-78]. In response, there is growing acceptance of the need to meaningfully engage users throughout the design, implementation, and evaluation of mental health interventions [79,80], service improvements [81,82], and priorities and processes for research [83-86]. Human- and user-centered design approaches to these health innovations, which prioritize user needs and iterative development processes, have been shown to maximize their usability and satisfaction, and support the translation of evidence-based innovations from research to practice [87-89]. Similarly, co-design approaches, which incorporate multiple stakeholders, including users with LE, can help to maximize the relevance and benefits of health innovations for those users [90,91], promote behavior change [92], support empowerment, uptake, and engagement [93,94], and reduce waste [95,96]. However, although emerging research suggests user-centered and lived-experience informed co-design is well suited to developing and improving cognitive assessment tools, this remains rare [78,97-99]. Additionally, though usability and user satisfaction are recognized as critical to long-term uptake and translation of health innovations, few gamified assessments or interventions are evaluated on this basis [42,88,100].

Our aims for this study were to redesign the CIS and to evaluate user satisfaction, using a collaborative sequential mixed methods co-design framework, informed by both evidence and experience [89,101]. Specifically, our researchers synthesized research evidence and stakeholder feedback to identify the need to reimagine the CIS and to ensure it retained its validated scientific structure (ie, the same cognitive constructs would be assessed in the same manner). Individuals with LEs would co-design guiding principles and ideas for game concepts and features through focus groups, alongside technology experts and researchers (refer to Ideation and Design Phase section), and would leverage their LEs to test and evaluate the new product (refer to Evaluation Phase section). Clinicians would cofacilitate focus groups and provide guidance to researchers to support the psychological well-being of participants. Technology experts would produce the new games through an iterative agile development process in close collaboration with researchers (refer to Production Phase section).

## Methods

### Overview

We used a sequential mixed methods co-design approach to reimagine the CIS assessment tool [102,103]. We adopted a pragmatic paradigm to achieve our objectives [104], centering users with LE as key informants at critical stages of the design process to maximize usability and satisfaction with the assessment tool [87], integrating researcher perspectives to retain the mechanistic structure of the original CIS games [101], and acknowledging the resource-limited context [89,92]. As such, our methodology adopts a framework that combines user-centered approaches to specifying requirements and evaluating the design, with rapid, low-cost production [105-107]. Here, we define co-design as a participatory process in which stakeholders with various forms of relevant expertise collaborate to address a problem concerning a system or tool [108-110]. This methodology was driven by an ethical commitment to elevate the voices of individuals with LE who will be most impacted by our research [111,112]. Furthermore, the research team values the deep experiential insights and “practical knowing” that can be accessed through participatory inquiry and researcher reflexivity, while also endorsing a critical realist perspective that values quantitative measurement of psychological phenomena [113]. Given this standpoint and our research goals, we adopted a thematic analysis (TA) approach to qualitative data analysis, a family of analytical methods that centers participant perspectives, and that acknowledges the active role of the researcher [114-116].

Details of research designs, participants, data collection, and data analysis for each phase (ideation and design, production, and evaluation) are reported below according to American Psychological Association mixed methods journal article reporting standards [117].

### Ideation and Design Phase

#### Research Design Overview

The qualitative ideation and design phase aimed to specify user requirements and produce a detailed design brief to guide the reproduction of the CIS. This was conducted through 2 focus group co-design sessions, which incorporated individuals with LEs of EDs and general mental health concerns, researchers, clinicians, and technology professionals. Our LE co-design approach in this phase was informed by the Australian Eating Disorder Research and Translation Center, which funded the production and evaluation phases of this project [86,118], and by user-centered design principles for eHealth and serious games [87,107,119].

#### Ideation and Design Study Participants

##### Researcher Description

The research team includes a PhD candidate with experience in qualitative and quantitative research, psychological assessment, and software development life-cycle (EC), a clinician-researcher with experience in EDs, addiction, and trauma (AA), a psychologist (LH), and a researcher with experience in qualitative and quantitative research and measurement development (CM). Noting researcher reflexivity and self-reflection as a critical aspect of reflexive TA [120-122], the research team reflected on their positionality and perspectives relevant to the research objectives and aimed to faithfully represent the user requirements and design preferences identified from this phase. Researcher identity and positionality statements are provided in Table S2 in Multimedia Appendix 1.

##### Participants

A total of 7 individuals participated in the focus groups. Participant characteristics are reported in Table 1. While there is no universally agreed-upon sample size for TA research [123,124], this sample size was deemed practical to manage [125] and sufficient to achieve the research goals based on previous research [78]. Importantly, we aimed to produce one acceptable solution to our identified research problem, rejecting the concepts of a single correct solution or “data saturation,” which do not form part of TA approaches to qualitative inquiry [121,126].

**Table 1.** Lived experience design-phase participant characteristics (n=7).

Demographic characteristic	Value
Age (y), mean (SD; range)	27.59 (7.03; 19‐40)
BMI (kg/m^2^), mean (SD; range)	23.70 (9.68; 13.5‐38.4)^a^
Race or ethnicity, n	
Caucasian (White Australian or European)	5
Asian	2
Gender identity (self-identified), n	
Cisgender woman	4
Cisgender man	1
Transgender woman	0
Transgender man	1
Nonbinary or gender diverse	1
Current eating disorder diagnoses, n	
ARFID^b^	0
Anorexia nervosa	2
Binge eating disorder	1
Bulimia nervosa	0
EDNOS^c^ or OSFED^d^	1
None	3
Historic eating disorder diagnoses, n	
ARFID	0
Anorexia nervosa	2
Binge eating disorder	0
Bulimia nervosa	2
EDNOS or OSFED	1
Other lifetime psychiatric diagnoses, n	
ADHD^e^	1
Anxiety^f^	4
Autism	0
BPD^g^	1
Depression	7
OCD^h^	2
Other	0

a Two design-phase participants chose not to report their weight.

b ARFID: avoidant or restrictive food intake disorder.

c EDNOS: eating disorder not otherwise specified.

d OSFED: other specified feeding and eating disorder (including atypical anorexia, and BED/BN of limited frequency or duration).

e ADHD: attention-deficit/hyperactivity disorder.

f Anxiety disorders (including but not limited to generalized anxiety disorder, panic disorder).

g BPD: borderline personality disorder.

h OCD: obsessive-compulsive disorder.

##### Researcher-Participant Relationship

There was an established relationship between the research team attending the focus groups (AA, EC, and LH) who had collaborated on the development of the original CIS [3]. There were no preexisting relationships between the research team and any of the participants. Ethical concerns relevant to this research context included the unequal power relationship between researchers and participants. In addition to informed consent and ethical review procedures, psychologist, LH, attended the focus groups with the expressed role of supporting participant well-being.

### Participant Recruitment and Selection

We recruited participants aged 18‐40 years who had current or past diagnoses of any ED. For safety and compliance, exclusion criteria were currently experiencing psychotic symptoms, bipolar disorder, and suicidality. Additionally, we excluded individuals who had previously completed the CIS. Participants were recruited using Meta advertisements, existing participant databases, and through local and national ED organizations (eg, InsideOut Institute, Australia & New Zealand Academy of Eating Disorders, and Scientific Work in Anorexia Nervosa & Other Eating Disorders).

**Figure 1.**
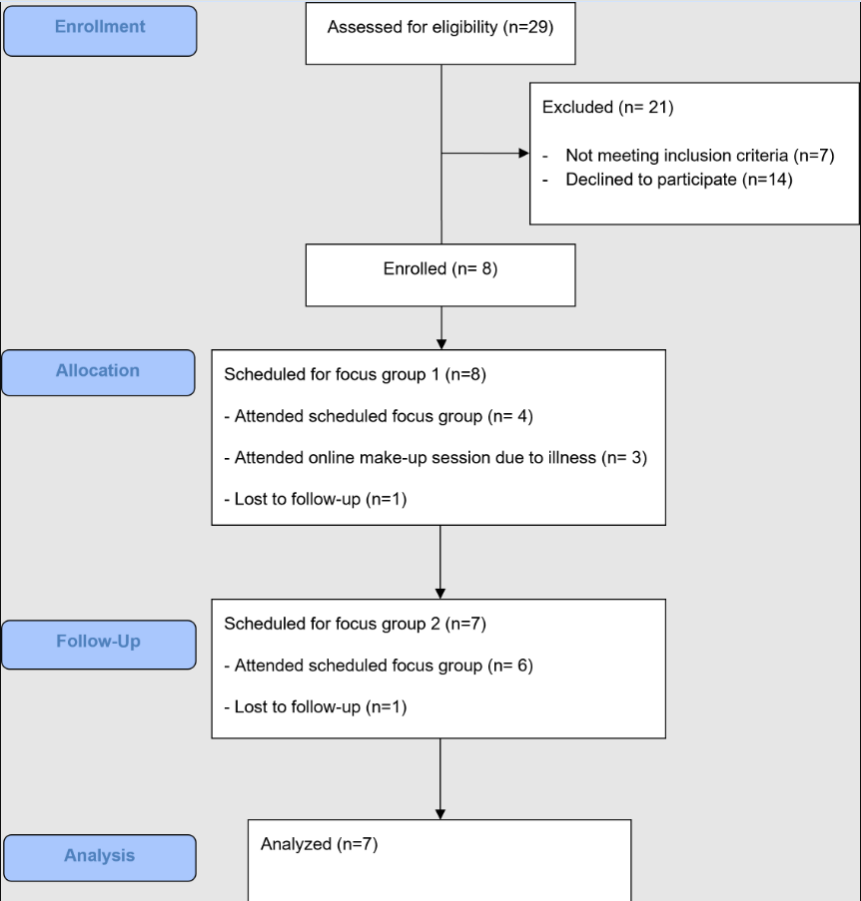
Design-phase participant flowchart.

Of the 29 who expressed interest in the study, 22 met eligibility criteria. A total of 7 participants were selected and attended the first focus group, with 6 participants returning for the second focus group (Figure 1). Focus groups were held in person at Monash University between June and July 2023. Participant characteristics are reported in Table 1.

The figure depicts a flowchart of participants who were enrolled, screened, allocated, and completed the ideation and design phase of the CIS redevelopment project from April to July 2023.

### Ideation and Design Data Collection

Participants provided informed consent and completed screening and enrollment questionnaires using REDCap (Research Electronic Data Capture) [127,128]. Eligible individuals were contacted to confirm eligibility and be briefed on focus group procedures. Participants attended 2 focus group sessions, scheduled 1 month apart, which were cofacilitated by EC and AA, and attended by a game development professional and a psychologist (LH). Each focus group lasted 3 hours.

Participants were asked open questions to generate ideas for a gamified cognitive assessment tool that should appeal to diverse audiences with EDs and commonly co-occurring conditions (refer to section S4 in Multimedia Appendix 1 for focus group guides). To avoid influencing ideas, participants were not shown visuals depicting the original game. Instead, brief details of the CIS’s general purpose, structure, and basic schematics were shared. A brief feedback survey collected their group and research experiences, such that improvements could be made for the second session (section S4.2 in Multimedia Appendix 1) [129-131]. In the second focus group, we honed design concepts and features and clarified key topics of conversation from the first session. Following the second session, participants were again invited to complete a brief survey to evaluate their experiences. Throughout these sessions, the facilitators, psychologist, and technology professional engaged in active discussions and whiteboarding with participants, prompting iterative review, expansion, and refinement of ideas. Sessions were audio recorded using Echo360 or Zoom software, and transcripts were redacted to protect anonymity.

### Ideation and Design Data Analysis

Data were analyzed according to the 6 stages of reflexive TA by Braun and Clarke [114]. An inductive approach was used to explore participants’ ideas and perspectives, while acknowledging the positivist, cognitive-behavioral perspective inherent in psychological assessments and the constraints imposed by the existing validated task structure. Reflexive TA further acknowledges that themes are actively generated in a subjective process, whereby the researcher interprets meaning from participant statements [126,132]. Final transcripts were coded by EC using NVivo 14 (Lumivero) [133].

In the first stage of reflexive TA, *deep familiarization*, transcripts and recordings were reviewed. The second stage involved *initial coding*, whereby an open coding approach to label segments of text within the data was used. In stage 3, *generating themes*, codes were grouped into categories to generate the main themes. Stage 4 involved *reviewing themes* to ensure a rich understanding of the data, whereby AA, LH, and CM were consulted to provide feedback on the drafted themes and possible interpretations. Themes were further refined in stage 5, *theme finalization*. Finally, stage 6, *interpreting and reporting*, involved the research teams’ continued revision of the codes and themes during the write-up phase.

### Production Phase

#### Research Design Overview

The production phase involved implementing the design ideas and guiding principles defined in the Ideation and Design Phase section. Here, we engaged *Noble Steed Games* (NSG) as an expert partner to design and produce the revised CIS. The new CIS was produced in an agile development process, involving 6 week-long iterative and collaborative sprints between NSG and the research team between March and April 2025. This approach was informed by guidelines and case studies concerning the pragmatic integration of user-centered design and LE co-design principles with the challenges of producing highly technical solutions that require expert knowledge, within a context of limited time and resources [88,92,105,106].

#### Production Participants

The production team involved 4 members of the NSG team, alongside the first author (EC). A play testing team was formed to assess usability and the extent to which the new CIS achieved user requirements and research objectives [119]. This team involved EC, CM, 3 student researchers, and 4 members of the AVG research group. All play testing members were familiar with LE co-design guiding principles and design ideas, and with the original CIS.

#### Production Procedures

NSG used CIS *Choices in the Wild West*, co-designed guiding principles, and cogenerated design ideas to inform their production of the new version of the CIS, referred to as *Papillon Park* (section S4, Multimedia Appendix 1). For the *Reflection* mini-game, NSG sourced copyright-free images from Unsplashed and Pexels, which were processed by EC to standardize size, color, contrast, and luminance properties using the *magick* package in R (R Core Team) [134]. During each sprint, NSG produced a specific deliverable (eg, wireframes for each mini-game; scene, stimulus, and character art; and scripts and instruction text), which were reviewed and refined in consultation with EC. Once available, playable prototypes were deployed to secure Monash servers, such that game functionality and data capture could be tested. These prototypes were further refined in response to play testing feedback, for example, by modifying stimulus timing and on-screen positioning to ensure target constructs were being effectively assessed. Following completion of the agile production period, ad hoc updates and refinements were defined by EC and implemented by NSG in response to LE play testing and evaluations. No research data were collected or analyzed during this phase.

### Evaluation Phase

#### Research Design Overview

The evaluation phase aimed to explore user experience satisfaction and the extent to which the design of CIS *Papillon Park* met user requirements [87,119]. To achieve these goals, we adopted a convergent mixed methods design. The GUESS-18 questionnaire was selected as an effective tool for quantitative data collection [135,136] and analyzed using descriptive and inferential statistics [116,137,138]. “Thinking aloud” was selected as an appropriate method for qualitative data collection, whereby participants provide a verbal commentary while playing the games [139,140], which was analyzed using deductive and inductive cycles of TA. While this method departs from the “Big Q” paradigm described by Braun and Clarke [115], this approach allowed us to answer our research questions by structuring our analysis according to the user requirements defined during the ideation and design phase, and is well-suited to convergent mixed methods approaches [116].

#### Evaluation Study Participants

##### Participants

We sought to retain participants from the ideation and design phase while recruiting additional participants to expand our sample size and composition. We planned to divide this sample into 3 approximately equal groups to examine whether participation in the ideation and design phase, or the process of “thinking aloud” while playing, influenced subsequent quantitative evaluations of user experiences and satisfaction.

##### Participant Recruitment and Selection

**Figure 2.**
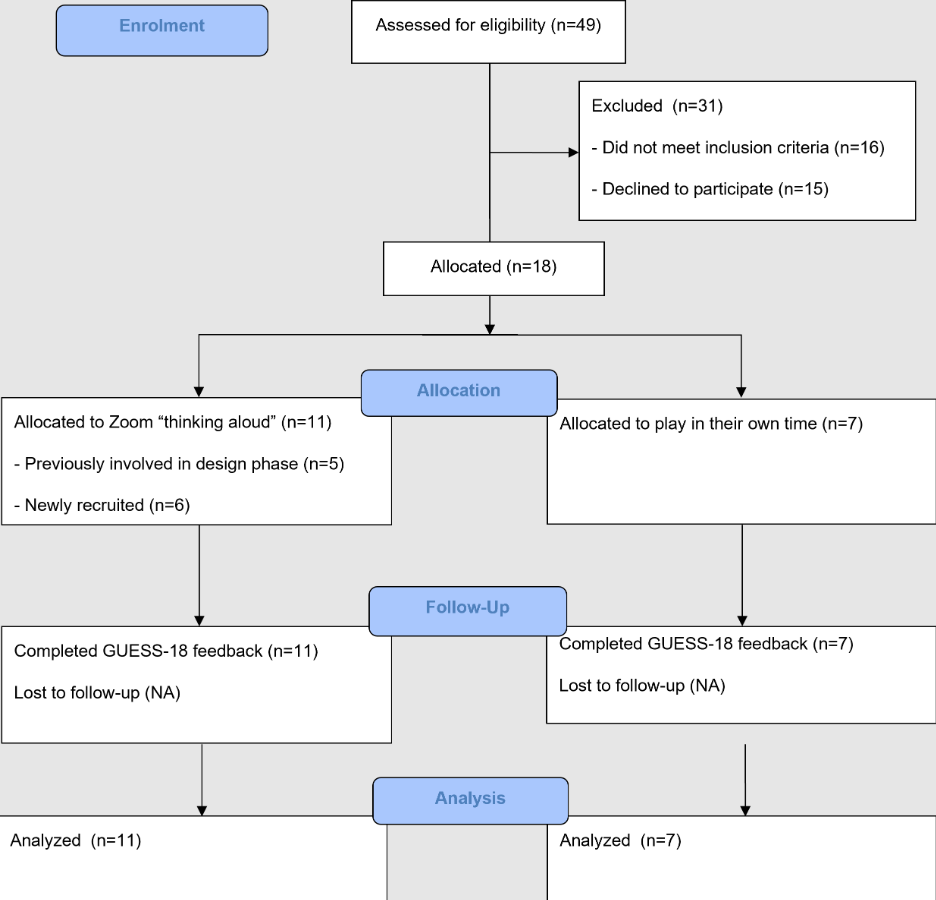
Evaluation-phase participant flowchart.

Participants who contributed to the ideation and design phase were invited to the present phase via email. Additional participants were recruited through Meta advertisements and existing participant databases. Identical inclusion and exclusion criteria used in the ideation and design phase were used. Of the total, 5 of the ideation and design phase participants returned for the evaluation phase. Of the 43 additional recruited participants, 27 met eligibility criteria, and 13 completed the study. All 5 participants from the ideation and design phase were invited to attend 1-hour Zoom sessions to play CIS *Papillon Park* while providing “thinking aloud” feedback. Newly recruited participants were split into 2 groups, such that 6 also completed Zoom “thinking aloud” sessions, and 7 played the game in their own time, according to their availability and preferences (Figure 2). Sessions took place in April 2025.

The figure depicts a flowchart of participants who were enrolled, screened, allocated, and completed the evaluation phase of the CIS redevelopment project from March to May 2025.

Characteristics of the 18 participants who completed the evaluation phase are summarized in Table 2.

**Table 2.** Lived experience evaluation phase participant characteristics (n=18; counts may sum to >18 due to cases with multiple diagnoses).

Demographic characteristic	Value
Age (y), mean (SD; range)	30.46 (6.56; 19‐42)
BMI (kg/m^2^), mean (SD; range)	23.78 (6.94; 15.6-42.7)^a^
Race or ethnicity, n	
Caucasian (White Australian, European, or North American)	11
Asian	7
Gender identity (self-identified), n	
Cisgender woman	12
Cisgender man	3
Transgender woman	0
Transgender man	1
Nonbinary or gender diverse	2
Current eating disorder diagnoses, n	
ARFID^b^	2
Anorexia nervosa	3
Binge eating disorder	0
Bulimia nervosa	1
EDNOS^c^ or OSFED^d^	2
None	10
Historic eating disorder diagnoses, n	
ARFID	1
Anorexia nervosa	1
Binge eating disorder	5
Bulimia nervosa	6
EDNOS or OSFED	4
Other lifetime psychiatric diagnoses, n	
ADHD^e^	6
Anxiety^f^	11
Autism	3
BPD^g^	1
Depression	8
OCD^h^	2
Other	2

a Four evaluation-phase participants chose not to report their weight.

b ARFID: avoidant or restrictive food intake disorder.

c EDNOS: eating disorder not otherwise specified.

d OSFED: other specified feeding and eating disorder (including atypical anorexia, and BED/BN of limited frequency or duration).

e ADHD: attention-deficit/hyperactivity disorder.

f Anxiety disorders (including but not limited to generalized anxiety disorder, panic disorder).

g BPD: borderline personality disorder.

h OCD: obsessive-compulsive disorder.

##### Sample Size, Power, and Precision

We did not perform an a priori power analysis. The target sample size of 21 participants was instead determined by the depth-oriented approach of the qualitative component of the evaluation phase, in accordance with established guidelines for this methodology and previous research [78,123].

##### Setting

Participants were provided with a link to access the CIS *Papillon Park* online and were instructed to use a computer with a keyboard (ie, not a phone or tablet). Participants were informed that the CIS would take approximately 40 minutes to complete and to complete the games in a single sitting. Participants completing “thinking aloud” sessions were provided with the link during their Zoom session, while those playing in their own time received it via email through REDCap. The 3 mini-games were presented in a randomized order, and server logs were used to confirm completion.

##### Quantitative Measures

The Game User Experience Satisfaction Scale, short version (GUESS-18) was used to gather quantitative feedback [135,136]. GUESS-18 is a psychometrically validated tool with 9 subscales that assess how various game attributes contribute to user satisfaction (Table S3.1 in Multimedia Appendix 1). Participants rated 18 items (eg, “I think the game is visually appealing”) on a 7-point scale from 1 (*strongly disa*g*ree*) to 7 (*strongly agree*) with a neutral midpoint of 4 (*neither agree nor disagree*). One item (“I feel bored while playing the game”) is reverse-coded. Subscale scores (1-7) are calculated by averaging numeric ratings for component questions, and a composite score (9-63) is obtained by summing subscale scores, with higher scores indicating greater user satisfaction. In this study, internal reliability for the composite scale was excellent (Cronbach α=0.93, Zumbo ordinal α=0.95). For subscales, refer to Table S3.2 in Multimedia Appendix 1.

After critically analyzing the alignment of the GUESS-18 subscale constructs to the co-designed guiding principles in the ideation and design phase (Table S3.3 in Multimedia Appendix 1), 2 items were developed to explore theme, *No Trigger Warnings Needed* (“I feel the games are appropriate for someone with lived experiences like mine,” “I feel the games are appropriate for someone with lived experiences like mine,” “I feel the games are triggering for someone with lived experiences like mine” [reverse coded]). Space for additional open-text feedback was provided at the end of the instrument.

### Evaluation Data Collection

Participants provided informed consent and completed screening and enrollment questionnaires online via REDCap [127,128].

#### Qualitative Data Collection

Of the 18 participants, 11 confirmed their availability for “thinking aloud” sessions to take place via Zoom [139]. During the session, participants were asked to access CIS *Papillon Park* online and provide a running commentary of their thought process as they proceeded through the game. Participants were advised that the facilitator (EC) would simply observe unless they became quiet, in which case a prompt would be provided. “Thinking aloud” sessions were audio- and video-recorded and transcribed using Zoom.

The remaining 7 participants preferred to complete CIS *Papillon Park* in their own time and were invited to provide qualitative feedback using a free text box in the online feedback questionnaire.

#### Quantitative Data Collection

After completing the CIS *Papillon Park,* participants were directed to complete the GUESS-18 questionnaire and the additional 2 items concerning the appropriateness of the tool for individuals with lived and living experiences of EDs and general mental health concerns (section S3.1 in Multimedia Appendix 1).

### Evaluation Data Analysis

#### Qualitative Data Analysis

Transcripts were reviewed for accuracy and uploaded to NVivo 15 for analysis [141]. First author, EC, undertook deep familiarization with the data. Data were coded to first identify technical or usability issues that could be rapidly resolved by NSG (eg, statements concerning misunderstandings of game instructions). EC then conducted deductive and inductive cycles of TA [116]. The co-designed guiding principles were used as a coding framework for deductive TA, with statements coded for insights into the extent to which the new tool met these user requirements. Data were then revisited with an inductive lens, seeking further insights into user experiences that had not been captured during deductive analysis. Finally, EC and CM *reviewed* codes, initial themes, and interpretations for clarity and narrative consistency. Themes continued to be *refined* during the *write-up and reporting* phase.

#### Quantitative Data Analysis

GUESS-18 responses were scored using Microsoft Excel and analyzed and visualized in R Studio [142]. There were no missing data. Means and SDs were calculated for total user satisfaction and for each of the 9 subscales according to published guidelines [135,136]. Exploratory omnibus ANOVA tests were used to examine group differences in GUESS-18 overall and subscale satisfaction between the 3 groups of participants (ie, designers, newly recruited “thinking aloud” participants, and those who completed the games in their own time).

#### Data Integration

The integration of qualitative and quantitative feedback data was intended to provide greater value than each alone [143]. Qualitative evaluation using a psychometrically sound tool facilitates comparison with other serious games and gamified psychological assessments, which is challenging when using only qualitative data, but lacks depth, and does not distinguish between the 3 component games of the overall tool. Conversely, qualitative data allowed us to understand *why* users felt satisfied or dissatisfied with particular elements of their experiences, and the variability in their experiences between the 3 mini-games. To achieve these goals, we aimed to synthesize and interpret qualitative and quantitative data at individual, group, and sample levels, and present an integrated narrative of our findings in the results section [137].

### Ethical Considerations

Ethical approval for all research procedures was provided by Monash University Human Research Ethics Committee (reference 29393), and all research participants provided informed written consent for the data collection and analysis described herein. Data were deidentified to protect participant privacy and confidentiality. No participants can be identified by the information included within the main paper or supplementary file, where participants are referred to individually (eg, in quotations), pseudonyms have been used. Participants with LE were compensated Aus $60 (approximately US $44) for each focus group (or Aus $20 [approximately US $15] for Zoom make-up sessions) and Aus $40 (approximately US $29) for the evaluation phase.

## Results

### Ideation and Design Phase Results

#### Overview

In total, three overarching themes were defined with a total of 9 subthemes that describe guiding principles for redeveloping the CIS: (1) aesthetic appeal and a progressive story create an immersive user experience; (2) achieving individual and collaborative goals fosters player motivation and purpose; and (3) relatedness, variety, and autonomy: fulfilling key psychological needs enhances user engagement. The meanings of these themes and their subthemes are explained below, with illustrative quotes provided in Table 3.

**Table 3.** Summary of co-designed guiding principles and illustrative focus group quotes.

Theme and subtheme	Illustrative quotes
Aesthetic appeal and a progressive story create an immersive user experience	
1.1 An interactive journey	“So, there’s a narrative, overarching kind of structure that you have to follow, and the entire story is just the mission.” [Ang] “I like collecting things along the way … or like, just like interacting with objects in a way that doesn’t necessarily add anything to your character or development, or anything, but just little interactions that are there.” [Erin]
1.2 Whimsical worlds have broad appeal	“There are a lot of like popular games that take a lot of elements of the real world, but then they adapt it into like their own art style, I guess, like aesthetic. It’s just, very NICE to look at. And yeah, I guess the animations and the storyline, it’s just very relaxing.” [Georgie] “I guess, I just find them very happy and appealing to look at … whimsical, is kind of the word that comes to mind.…. I guess it’s a bit childish, but it’s like happy childhood. It’s sort of like happy childhood memories. Yeah, it’s got that whimsical nature to it, but it still blends in a bit of reality.” [Fiona] “I think when I’m playing games, quite often, I would be doing it to kind of get out of the real world. I think a lot of people who just use games as like a form of escapism.*”* [Chloe]
Achieving individual and collaborative goals fosters player motivation and purpose	
2.1 Growing and “leveling up”	“I really like being able to level up and acquire certain skills.” [Erin] “With the experience, then you sort of level-up… you can purchase items that help you … or certain sorts of clothing, hairstyle, something like that.” [Ang]
2.2 A prosocial purpose	“For me, that’s a quest … you find the person or the clues to save humanity.” [Bethany] “Oh yeah! Rescuing the towns people! That would be so cute!” [Fiona] “I was thinking of growing something, potentially.” [Dylan] “Yeah, whether it’s a garden or a forest. Just … more plant life, the better you do.” [Dylan] “I feel like it’s all in a game. You know that this is virtual. It’s not real to me. I think others probably feel differently though.” [Ang]
2.3 Positive insight	“I was thinking perhaps keeping it more towards, say, benefit versus less benefit as opposed to danger versus no danger.” [Dylan] “I like that it’s not catastrophic, you know? You don’t lose entirely.” [Ang] “Interpretation is more helpful. A leader board is a terrible idea.” [Bethany] “Because it just has that social comparison aspect.” [Erin] “Yeah, even though I’m trying, everyone else is doing so much better than I am.” [Bethany]
Relatedness, variety, and autonomy: fulfilling key psychological needs enhances user engagement	
3.1 Batman needs his Alfred	“You’re Batman, and you need your Alfred.” [Bethany] “I think belonging to a group does enhance a game, whether it’s a community or a gang or whatever. It creates more responsibility. And I really like to be able to get to know the people in your community or team, or whatever, and them all having their own little personalities and stories.” [Erin] “A diverse set of characters is more engaging, and each of the interactions adding to the storyline, adding details to this world. I feel that would make it more engaging.” [Georgie]
3.2 Variety is the spice of game-life	“I think, depending on how often in the game this is going to occur, [it’s important] for it to not be the same every time …. I think I’d get a bit annoyed.” [Erin] “I like games with time limits …” [Chloe] “Whoa, so stressful!” [Ang] “Yes but keeps it really interesting.” [Chloe] “Where it’s not too difficult where it’s frustrating, but it’s not too straightforward.” [Georgie]
3.3 Choose your own adventure	“I was thinking of a Harry Potter game I used to play when I was a kid, and you could, choose different wands. And that would give you different abilities.” [Chloe] “Yeah, Like different characters, different paths? Yeah. It’s like the first game that I ever played was Pokémon. You choose the different sort of Pokémon, right? And then based on the different type, you have different abilities and so on.” [Ang] “I like changing the environment … in terms of customisation.” [Bethany]
3.4 No trigger warnings needed	“The main thing that I was thinking about was in some games you just get a little bit too much freedom with customizing your character. Most of the time people just end up creating, I guess what they wish they looked like. But then it just gets a bit triggering or problematic once you get to adjust heights or body shape and size and stuff. So, yeah, I feel too much customization of characters in games would be triggering.” [Georgie] “I think diversity in size, shape, all that - that’s a big one. Size and shape in video games, there’s not much diversity. So, I think that’s really important.” [Erin] “I think there’s going to be a lot less issues around inclusivity and stuff if you’re just playing from behind the camera.” [Erin] “I think, if you’re the one looking in, you’ve got less issues, with things like gender representation. If you’re representing other people or characters, they need to be diverse.” [Bethany]

#### Theme 1: Aesthetic Appeal and a Progressive Story Create an Immersive User Experience

##### Subtheme 1.1: An Interactive Journey

Participants described a preference for first-person game-play and a sense of advancement and forward momentum within a cohesive narrative structure. For example, the playing character would proceed through a journey or quest across a series of tasks, completing these as missions toward their overall goal. Participants also agreed that the playing experience would be improved by exploring varied environments as they progressed through the story.

##### Subtheme 1.2: Whimsical Worlds Have Broad Appeal

Participants described wanting the games to have an appealing art style, with characters and environments that are cartoonish, simple, and colorful, and a playing experience that is gentle and accessible. They consistently drew on examples such as Nintendo’s *Super Mario* and *Animal Crossing*, and there was consensus that these features appeal to “gamers” and “nongamers” alike. Participants described a sense of escapism in these whimsical worlds as a key attraction to gaming. They also described the enjoyment of completing relatively simple or familiar tasks that have been rendered funny, quirky, surprising, or novel by the whimsical and cartoonish environment, or by the abilities (or lack thereof) of their playing character.

### Theme 2: Achieving Individual and Collaborative Goals Fosters Player Motivation and Purpose

#### Subtheme 2.1: Growing and Leveling Up

Participants discussed the most meaningful goals and rewards to be gaining knowledge, skills, and expertise. These gains might contribute directly to the overall game goal, achieve personal growth, or be used to augment the character or their environment.

#### Subtheme 2.2: A Prosocial Purpose

Participants resonated with having an overall objective that helped others, although one participant felt this was less important. Participants agreed, however, that rewards should be nonmonetary, and that in-game “currency” should be instrumental to achieving their prosocial goal, rather than “capitalistic.” Examples included rescuing townspeople, growing plant life, or taking care of realistic or mythical animals.

#### Subtheme 2.3: Positive Insight

Participants discussed that leaderboards and comparisons to other players’ performance could be motivating; however, consensus was reached that feedback should avoid social comparison. The participants agreed that poor performance should be “safe,” such that no in-game characters would be harmed or imperiled if the player gained relatively little in-game currency or points. As much as possible, outcomes should be neutral or positive and avoid damage or significant losses, and feedback should be normalizing and supportive.

### Theme 3: Relatedness, Variety, and Autonomy: Fulfilling Key Psychological Needs Enhances User Engagement

#### Subtheme 3.1: Batman Needs His Alfred

Participants felt games played within a mental health context should incorporate positive in-game relationships. One example was through a constant supporting presence of a sidekick, or as one participant stated, “*You’re Batman, and you need your Alfred.*” In addition, participants felt the playing character could be part of a virtual team or be depicted engaging positively with nonplayable characters (NPCs) from the broader community. This subtheme, therefore, is closely related to pursuing a prosocial purpose.

#### Subtheme 3.2: Variety Is the Spice of Game Life

It was emphasized that incorporating as much variety as possible within the constraints of the task structure would be key to maintaining player motivation and would prevent the player experience from becoming monotonous. Participants also felt an appropriate level of difficulty and challenge to be important; while too little can be boring, too much becomes stressful and a barrier to engagement.

#### Subtheme 3.3: Choose Your Own Adventure

Participants requested the ability to make customizations and to interact with or construct the environment around them. These experiences were felt to create richness and immersion in games, as well as providing a sense of autonomy that fosters active engagement.

#### Subtheme 3.4: No Trigger-Warnings Needed

To maintain psychological safety, the participants agreed on the importance of avoiding the ability to customize or alter human forms and of diversity in the depiction of human and human-like characters. Avoiding stimuli or gameplay relating to food and eating, body image, alcohol, drugs or gambling, violence or crime, and gendered, racial, or cultural stereotypes was also discussed as critical for individuals who share their lived and living experiences.

In addition to the themes described above, the focus groups generated many specific ideas for each of the component games, which were summarized and communicated to the game developers (section S4 in Multimedia Appendix 1). Importantly, the focus group participants also evaluated their ideation and design phase experience positively (section S4.2 in Multimedia Appendix 1).

### Production Phase Results

**Figure 3.**
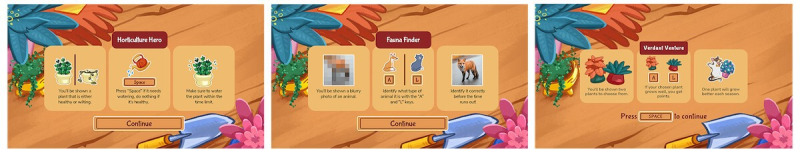
Introductory screens for *CIS Papillon Park* component mini-games: Horticulture Hero (attentional control), Fauna Finder (reflection), and Verdant Venture (feedback monitoring/shifting).

To achieve the co-designed themes while maintaining the validated structure of the original CIS*,* our game development partner produced the CIS *Papillon Park* with 3 component mini-games featuring a cohesive community garden theme. Cognitive Impulsivity is assessed as users proceed through *Horticulture Hero* (attentional control), *Fauna Finder* (reflection), and *Verdant Venture* (feedback monitoring or shifting), which can be presented in set or randomized order. Each game occurs within a unique garden environment and begins with a brief practice mode before the scored blocks (Figure 3).

The figure depicts 3 colorful cartoon images of a greenhouse workbench, each overlaid with 3 images from the mini-games, explaining their goals and response keys.

**Figure 4.**
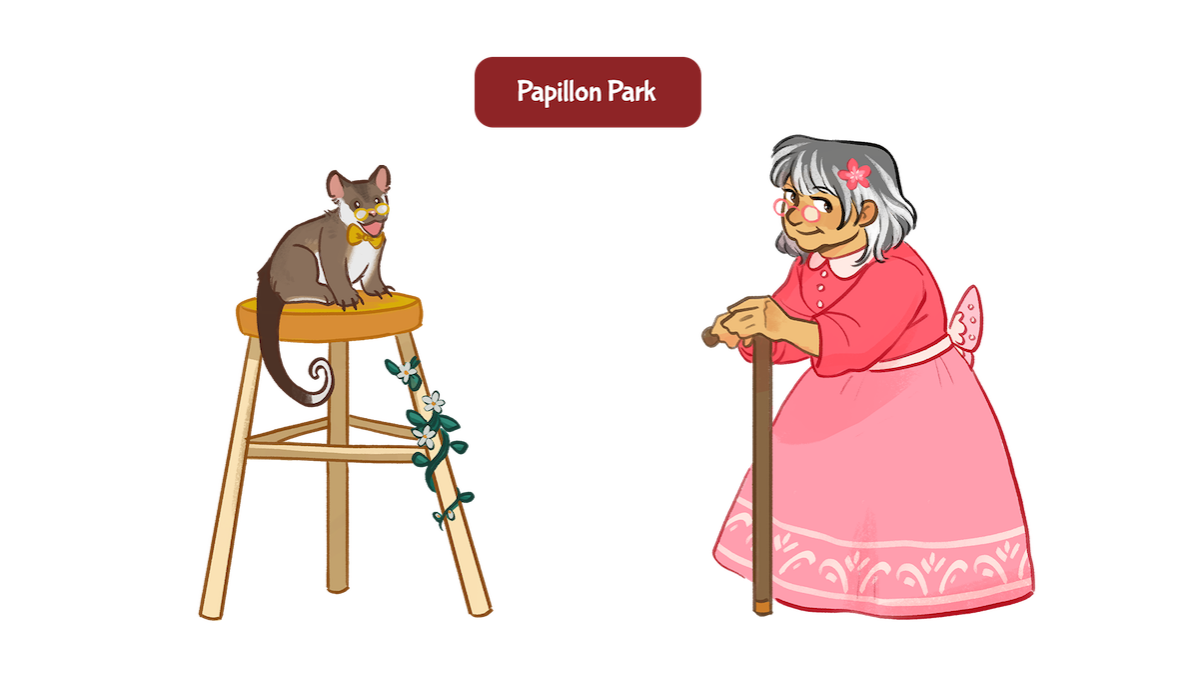
Winston and Grandma Pixie.

Users are led through the games by 2 virtual helpers—“Grandma Pixie” and “Winston” (Figure 4), and interact with diverse NPCs who reinforce their progress and the positive impact it has on the community. At the conclusion of each mini-game, users receive their total points and are offered a choice between 2 rewards to decorate the community garden. Their progress is visually depicted with their chosen reward and new growth.

The figure depicts an Australian ringtail possum wearing glasses and a yellow bow tie seated on a stool, alongside an older adult woman dressed in pink and leaning on a wooden walking stick. These virtual helpers provide instruction, performance feedback, and companionship to the user throughout *CIS Papillon Park*.

### Evaluation Phase Results

#### Qualitative Outcomes

Qualitative evaluations were consistent with the 3 superordinate themes and subthemes that participants described during phase 1. The meanings of participant evaluations are described below, with illustrative quotations provided in Table 4.

**Table 4.** Summary of thematic evaluations and illustrative quotes.

Theme and subtheme	Illustrative quotes
Aesthetic appeal and a progressive story create an immersive user experience	
1.1 An interactive journey	“Overall, I quite liked the flow of the storyline and the characters that I interacted with…. It’s a very well-developed experience.” [Liza] “I like that it’s got a little ongoing story to kind of …. I feel like it like with that, and mixing things up, it kind of adds to that gamified element by like giving you something to work towards.” [Jay]
1.2 Whimsical worlds have broad appeal	“I found the graphics and background music/side effects of the game to be very calming and immersive.” [Lily] “I like the way that this is designed. I’ve done similar things to this before, where, um, I guess they’re assessing a similar sort of thing where it’s hit the space bar when a particular number comes up on screen. But I think the way that this is … visually it’s a lot more ... interesting, which is nice. It’s got nice bright colors, which makes it a little bit more fun to look at, versus your kind of black and white numbers on a screen.” [Jay]
Achieving individual and collaborative goals fosters player motivation and purpose	
2.1 Growing and “leveling up”	“You probably get a little better at this game as it goes on … as you get more familiar with it.” [Tom] “The ‘practice’ rounds of each game helped allay any anxiety I had about learning how to play the game and I found them all quite easy to pick up quickly.” [Olivia] “I would say that overall, it’s an interestingly positive experience, because, firstly, it’s not a steep learning curve. Not at all. I like and how simple it is, and how easy it is to pick up.” [Ang] “It did make me disheartened to not be able to figure out the pattern for this game and made me question whether I wasn’t smart enough.” [Millie]
2.2 A prosocial purpose	“[The NPC’s] were great. I think they were all very likable.” [Jess] “Maybe more people will come and visit and bring me [Winston] presents … I want that!” [Grace] “I’m wondering if these random visitors are gonna come back into the game, - if they like if they play a role, or if they are just … there.” [Erin] “Grandmother’s a bit patronizing, isn’t she?” [Bethany]
2.3 Positive insight	“The sound effects when I got answers right/wrong were very cute … and I felt a tinge of sadness when getting an answer wrong, but I didn’t inherently feel upset for the rest of the game.” [Liza] “I wouldn’t say it’s easy not to beat yourself up about it …. but I like how low stakes this is.” [Ang] “I was left wondering how I had performed versus other people playing the game in this study.” [Liza]
Relatedness, variety, and autonomy: fulfilling key psychological needs enhances user engagement	
3.1 Batman needs his Alfred	“I think the art is a really good motivator because you get to see little Winston and Grandma Pixie … Who doesn’t like a happy Winston?” [Alex] “I like his happy sound … if you’re happy, I’m happy.” [Grace] “That’s three 200’s in a row, Winston. I hope you’re happy, little buddy.” [Ang] “I’m gonna end up in the negatives. Sorry, Winston.” [Lily]
3.2 Variety is the spice of game-life	“The [horticulture hero] game was interesting and I liked that the plants changed each round.” [Liza] “It’s nice to see the variety of points change this time” [Alex] “I enjoyed the blurry animal guess game most - it wasn’t the same choices repeated and it got you to engage cognitively with time urgency.” [Millie] “I feel like the whole design of this game is just to test my patience!” [Jess] “[There were] too many rounds to make it to the next stage. It became quite tedious, boring and irritating.” [Jo] “If I was playing this game under normal circumstances, like at home, I might just turn it off, because it’s really long.” [Chloe]
3.3 Choose your own adventure	“Oooh, there’s a bird or a mushroom … I want the bird. (Gasps). It’s a bird fountain! Oh my gosh that’s so cool. OK, yes, I agree, wonderful choice, I know!” [Grace] “That’s cute … I like how you can customize it.” [Erin] “In the blurry game [Fauna Finder] … I felt I could actually engage and make a difference rather than something allocated/randomized.” [Lexi] “The first game was confusing as it was not clear what made each plant a successful choice [and why there wasn’t 100 points, but only − 100, 50 and 200] and made me think the success was randomized like a gambling game rather than anything indicative of your own performance and choices.” [Alex]
3.4 No trigger warnings needed	“I really like the characters around here, because I can see that a lot of thought’s gone into their design.... Very interesting gender-neutral choice.” [Ang] “It was nice that there was like a diverse range of different people that came in.” [Fiona] “The choice of animals - since you’ve got such realistic pictures - gorgeous for animal lovers, and they’re not generally one’s people get too icked out about - possibly the mice, but, like, if you were showing pictures of snakes, I wouldn’t be able to play.” [Bethany] “I wonder what would happen to somebody who had a phobia of frogs.” [Ang]

#### Theme 1: Aesthetic Appeal and a Progressive Story Create an Immersive User Experience

##### Subtheme 1.1: Interactive Journey

Perspectives concerning the game story, context, and environment were consistently positive. Participants enjoyed the overall story and community garden theme, and that each game took place within a different environment to provide variety and achieve the desired sense of progression.

##### Subtheme 1.2: Whimsical Worlds

All participants praised the art style and felt the visual and sound designs enhanced their playing experience. Participants described the designs as appealing, calm, and soothing, and as effectively “drawing players in.” They felt these designs were key to maintaining their motivation and drew positive comparisons to standard cognitive assessments they had experienced.

### Theme 2: Achieving Individual and Collaborative Goals Fosters Player Motivation and Purpose

#### Subtheme 2.1: Growing and “Leveling Up”

Participants described feeling that they were able to learn and improve their skills in positive terms. However, the degree to which participants endorsed this feeling varied across the 3 mini-games; the most positive statements related to Fauna Finder, which participants felt was more skill-based, and the more negative statements related to Verdant Venture, which participants tended to find frustrating.

#### Subtheme 2.2: A Prosocial Purpose

The community NPCs were found to be likable and appealing, and participants enjoyed seeing their achievements benefit Winston, their virtual companion. However, while some participants enjoyed interacting with the NPCs and learning that their performance was benefiting the community, others did not connect with this intended feature or found the NPC interactions to be protracted. Grandma Pixie’s communication style also inspired some more polarized responses.

#### Subtheme 2.3: Positive Insight

Participants felt the game created a gentle, low-stakes environment that still engaged them emotionally and cognitively—enough to care about their performance, but not enough to feel stressed—and felt their interactions with Winston effectively supported this. Participants provided more mixed feedback relating to the lack of context for point totals; some participants wanted to understand their performance in comparison to other players or the maximum available, while others appreciated that this was not a feature.

### Theme 3: Relatedness, Variety, and Autonomy: Fulfilling Key Psychological Needs Enhances User Engagement

#### Subtheme 3.1: Batman Needs His Alfred

All participants responded positively to Winston’s companionship. Participants commented that they were motivated to perform on behalf of Winston, engaging emotionally with his audio and visual performance feedback.

#### Subtheme 3.2: Variety Is the Spice of Game-Life

Participants reported high enjoyment, engagement, and motivation when stimuli, responses, and rewards were varied, and when they felt their behavior was consequential. As with *Growing and leveling-up* above, these experiences varied across the mini-games, with participants typically finding Horticulture Hero to be easy but boring, Fauna Finder to be the most purposeful and engaging, and Verdant Venture to be the most difficult, random, and confusing. Moreover, 2 participants felt the game was overall too long.

#### Subtheme 3.3: Choose Your Own Adventure

Although opportunities for customization were limited, the ability to select from available rewards and to see those rewards depicted as garden decorations was appreciated and enhanced player engagement and fulfillment.

#### Subtheme 3.4: No Trigger Warnings Needed

Finally, participants praised the diverse character depictions and “wholesome” gameplay features. They felt the production phase had been attentive to inclusivity and emotional safety, while also highlighting the need to consider individual sensitivities to the animals depicted in *Fauna Finder*.

### Quantitative Outcomes

**Figure 5.**
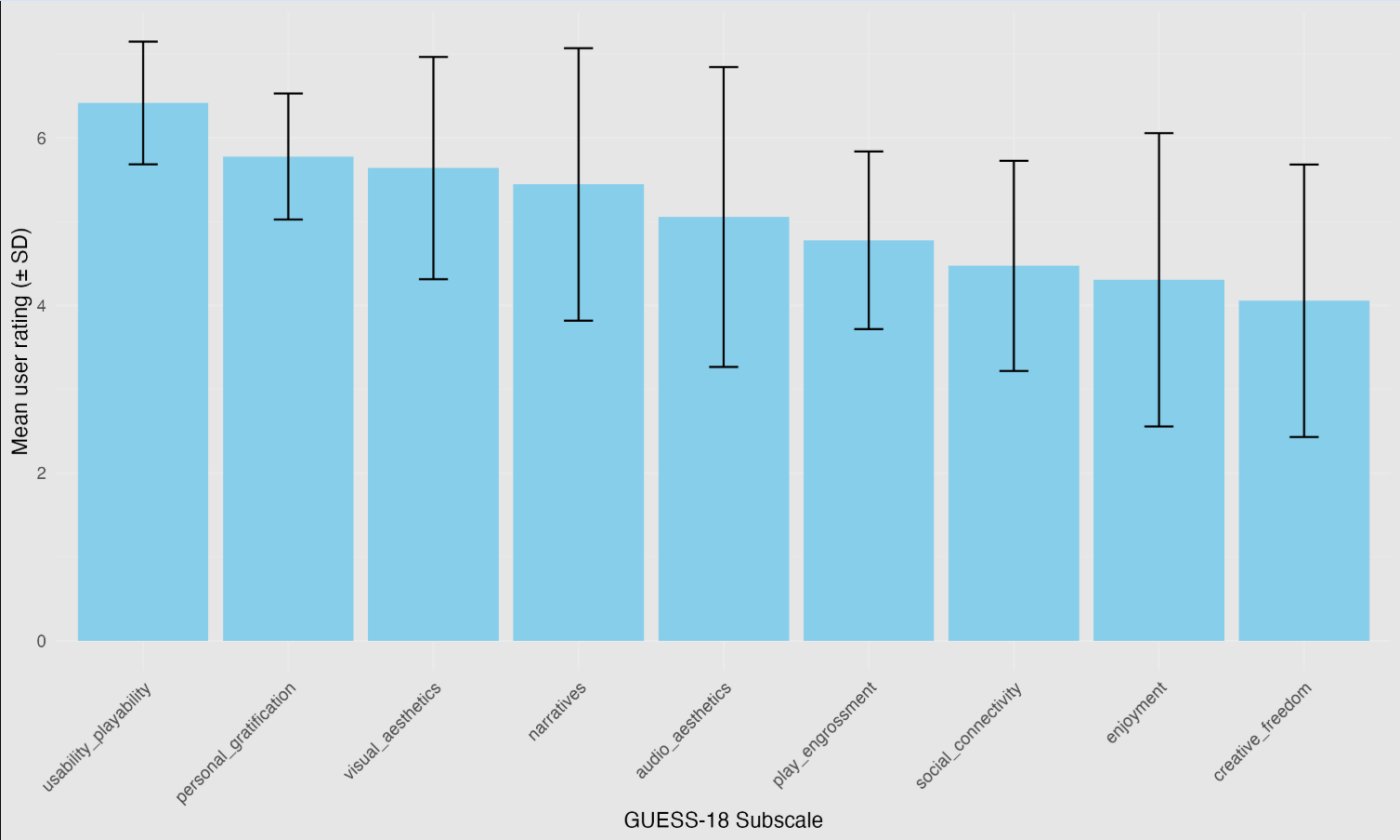
Game User Experience Satisfaction Scale, short version subscale ratings.

Evaluations of the games were overall positive, with a mean GUESS-18 composite score of 45.9 (SD=9.85; 72.9%, 57.3‐88.5), and subscale scores ranging from 4.1 (neutral) for “Creative Freedom” to 6.4 (agree-to-strongly agree) for “Usability or Playability” (Table 5; Figure 5). There were no significant differences in mean composite or subscale scores between the groups of participants who were involved in the ideation and design phase, those who completed the “thinking aloud” evaluation over Zoom, and those who evaluated the games independently (Table 5).

**Table 5.** Game User Experience Satisfaction Scale, short version user satisfaction survey results.

Measure	All (n=18), mean (SD)	Designers (n=5), mean (SD)	Zoom (n=6), mean (SD)	Own time (n=7), mean (SD)	Group differences (ANOVA)^a^	
					*F* test (*df*)	*P* value
Composite score^b^	45.9 (9.85)	47.4 (4.98)	42.4 (10.38)	47.9 (12.25)	0.55 (2, 15)	.59
Subscale scores^c^						
Usability or playability	6.4 (0.73)	6.7 (0.27)	6.3 (0.76)	6.4 (0.94)	0.52 (2, 15)	.60
Personal gratification	5.8 (0.75)	5.8 (0.76)	5.7 (0.88)	5.9 (0.75)	0.10 (2, 15)	.91
Visual aesthetics	5.6 (1.33)	5.8 (0.57)	5.3 (1.40)	5.8 (1.73)	0.22 (2, 15)	.81
Narratives	5.4 (1.63)	6.1 (0.82)	5.1 (1.77)	5.3 (1.98)	0.56 (2, 15)	.58
Audio aesthetics	5.1 (1.79)	5.5 (1.70)	4.9 (1.69)	4.9 (2.14)	0.19 (2, 15)	.82
Play engrossment	4.8 (1.06)	5.0 (0.35)	4.3 (1.21)	5.1 (1.21)	1.14 (2, 15)	.35
Social connectivity	4.5 (1.25)	4.6 (0.96)	3.9 (1.46)	4.9 (1.25)	0.94 (2, 15)	.41
Enjoyment	4.3 (1.75)	4.2 (0.76)	3.3 (1.72)	5.3 (1.89)	2.62 (2, 15)	.11
Creative freedom	4.1 (1.63)	3.7 (1.44)	3.8 (1.29)	4.6 (2.05)	0.55 (2, 15)	.59

a Achieved power for 1-way omnibus ANOVA tests was calculated as 0.74 using G*Power 3.1 (α=.05, sample n=18, 3 groups, and average effect size of *F*=0.76).

b Composite scores range 9‐63 with neutral midpoint 27.

c Subscale scores range 1‐7 with neutral midpoint 4; higher scores indicate greater user satisfaction.

The figure presents a bar graph depicting GUESS-18 subscale ratings for the full evaluation sample (n=18). Bars indicate mean ratings for each subscale, and error bars indicate the SD, arranged from the subscale with the highest user satisfaction (usability or playability) to the lowest (creative freedom).

Ratings for the additional questionnaire items concerning *No Trigger Warnings Needed* produced an overall mean score of 5.72 (SD 1.22). Ratings differed significantly by group (*F*_2,15_=7.64; *P<*.01) with significantly higher ratings in the 2 groups providing “thinking aloud” feedback than in the group playing in their own time (scoring and ANOVA outcomes for these items are provided in Table S3.4 in Multimedia Appendix 1).

### Integration

Qualitative and quantitative evidence converged to allow us to understand which design features and gamification elements were most effective in facilitating long-term user motivation and engagement. Specifically, both qualitative and quantitative data demonstrated that the “wholesome” environment, cohesive and progressive story, attractive visuals, and appealing audio created a positive emotional user experience, which in turn facilitated immersion and enjoyment.

Qualitative evaluations also complemented quantitative data by allowing users to explain *why* particular subscales were rated higher or lower. For example, the inclusion of virtual helpers, practice rounds, retries, a progress bar, a timer or speed element, and visual depiction of achievements were key to user motivation and underpinned the high levels of usability or playability and personal gratification. Similarly, qualitative feedback explained that perceiving the themes defined during co-design concerning desires for autonomy (*choose your own adventure*) and variety (*variety is the spice of game life*) to be lacking resulted in the lowest GUESS ratings for the “enjoyment” and “creative freedom” subscales. Importantly, qualitative feedback showed users perceived the 3 mini-games that form the CIS *Papillon Park* to vary according to these characteristics, as well as their perceived level of difficulty, such that user motivation and engagement varied significantly between each mini-game. This variability had not been captured in the quantitative user satisfaction ratings.

Qualitative and quantitative evaluations diverged in relation to the GUESS-18 ratings for social connectivity and the overwhelmingly positive appraisals of Winston. Further analysis indicated that lower ratings for social connectivity were associated with qualitative feedback that NPC interactions, and the game overall, were too long.

## Discussion

We aimed to redesign and evaluate the CIS, a validated gamified assessment battery of cognitive impulsivity, through a sequential mixed methods approach to user-centered co-design, agile game development, and user-centered evaluation [3,89,106]. We first defined LE user requirements, generating themes and subthemes that described overall guiding principles, and producing specific design ideas. The agile game development process resulted in a functioning suite of gamified assessments, the CIS *Papillon Park,* being deployed to secure Monash University servers. During LE evaluation, our qualitative analysis confirmed that the new tool largely met the co-designed user requirements. Quantitative ratings aligned closely, showing the CIS *Papillon Park* achieved strong overall user satisfaction according to the GUESS-18 composite scale, with strengths in usability, narrative, visual and audio aesthetics, and personal gratification.

Escapism into attractive virtual worlds featuring cohesive narrative storytelling and accessible gameplay, and a sense of personal growth and achievement, were identified as critical themes and key strengths of the gamified assessment tool produced in this study. Aesthetic and narrative characteristics are well-known to increase immersion and pleasure in gaming [144,145]. Furthermore, the gamification mechanisms we incorporated to foster personal accomplishment, including tasks with clear goals and rounds, points and performance feedback, in-game rewards, and visible depiction of achievements, are some of the most commonly used [59,65]. These characteristics were the most strongly endorsed in the qualitative and quantitative evaluations, confirming the new games to be broadly appealing and fulfilling, and reinforcing previous research demonstrating the value of aesthetics and gamification to user engagement and satisfaction with cognitive assessments [63,64,146]. These principles also align closely with the theoretical grounding of gamification [56,57,61], tapping into cognitive and emotional mechanisms to generate intrinsic motivation to engage with CIS *Papillon Park* [58,59]. In the context of cognitive assessments, engagement and intrinsic motivation serve critical instrumental purposes, encouraging uptake and continued, authentic effort, and thus valid assessment outcomes [45,46,62,66].

We also incorporated gamification elements that expand on those most used in mental health interventions, for example, the presence of virtual helpers [65]. Winston, Grandma Pixie, and the community NPCs reflected key ways in which our participants characterized mental health and positive functioning, including affirming interpersonal relationships and the pursuit of prosocial goals. Difficulties in these areas are well-documented features of EDs [147-152]. For example, socializing problems, social isolation, and loneliness have been shown to have a detrimental, bidirectional relationship with ED symptoms [152,153], while social connectedness may protect against ED onset and facilitate recovery [154-156]. As such, re-engaging with interpersonal relationships and meaningful life pursuits form common targets of harm reduction models of care and psychosocial recovery support for individuals living with EDs [157,158]. Within a gaming context, social interaction and collaboration toward mutual, prosocial goals are recognized as key not only to player engagement but also to realizing psychological benefits from gaming [58,59,66,159,160]. While real-time, multiplayer cooperation cannot readily be recreated within the context of cognitive assessment, evaluations suggested we had been successful in modeling meaningful collaboration and prosociality through reinforcing interactions with an endearing “side-kick,” although somewhat less successful in achieving this goal through interactions with members of a broader virtual community [161,162].

A key insight that emerged from the integration of qualitative and quantitative evaluation was the variability in user satisfaction between the 3 mini-games that form the CIS *Papillon Park* [143]. In total, 3 key elements distinguished user satisfaction—perceived autonomy and self-determination, variety, and level of difficulty. Our synthesized evaluation showed that gamification elements, including practice rounds, retries, a progress bar, and timer or speed, were key to achieving a level of difficulty that maximized user satisfaction [59,73]. Perceived lack of variety, autonomy, and creativity received lower quantitative ratings and inconsistent qualitative evaluations. These findings aligned closely with a previous study, where enjoyment helped people keep playing and maintained performance in easy games, while motivation facilitated performance and enjoyment in difficult games [163]. These findings reflect the inherent conflict between the repetition and standardization needed to achieve valid and reliable data in cognitive assessments, and user goals such as novelty and self-directed exploration that make games fun and engrossing, highlighting the difficulty of balancing the constraints of structured assessments with engaging game elements [62,63]. They also indicate that combining multiple gamification strategies, whereby elements enhance or compensate for one another, may be important in determining long-term user engagement [52,164].

User-centered co-design approaches are now commonly used to design mental health services and interventions leveraging web, mobile, and artificial intelligence technologies [48,74,78,99,100,165]. Less commonly, these approaches can be used to design and evaluate valid and reliable psychological assessment measures [42,88]. In developing the CIS *Papillon Park,* we took on board growing impetus to meaningfully incorporate LE perspectives throughout the research process [95,129]. Respectfully collaborating with individuals with lived and living experience in both design and evaluation is therefore a key strength of this study, underpinning its novel contribution to the field. Through evaluation, we were able to confirm user design priorities had been faithfully represented at all stages of the research process and were present in the final product [111,166]. Importantly, in addition to defining what to include in the new product, the co-designed guiding principles defined what *not* to include. It is important to note that some gamification elements might be harmful to certain populations, as exemplified by discussions concerning a leaderboard in this study [59]. Gamification is inherently experiential and subjective [54]; therefore, gamification and user-centered design work most effectively together, such that users define which gamification elements will be most safe and meaningful for them [167]. By following up on user-centered co-design with LE evaluations, we confirmed CIS *Papillon Park* maintained psychological safety for individuals with LEs of EDs and mental ill-health [168].

In terms of limitations, our co-design framework and qualitative methodologies meant we collaborated with relatively small groups of participants, and our quantitative evaluation analysis was somewhat underpowered. However, we nonetheless aimed that they would be representative of the target populations for the resulting assessment tool. We were therefore pleased to engage with groups that comprised varied current and historical experiences with disordered eating, that represented a broad range of experiences of commonly co-occurring mental ill-health, and that encompassed diverse identities and personal characteristics. These sentiments were shared by our participants, who endorsed the co-design groups and research processes as positive experiences, emphasizing their inclusivity [129,130]. This project was undertaken in Australia with participants of White European, White North American, and Asian ancestry; thus, its findings may be less relevant in other cultures. Our methodology incorporated various forms of expertise to achieve a practical goal of reimagining an existing cognitive assessment tool [101,104,116,169]. As such, we incorporate key principles of qualitative research but depart from certain canonical aspects of “Big Q” approaches [115,122]. Further methodological limitations include the challenges of integrating user-centered co-design and agile development in a resource-limited context [89,92,105,106]. In terms of the quantitative results, our ANOVA analyses were slightly underpowered, such that there is an elevated risk that we failed to detect differences in user satisfaction between groups.

Ultimately, this project intends to enhance the impact and reach of the CIS by facilitating its potential for clinical translation and adoption by end users [170]. Validated tools with streamlined administration and analysis pipelines that are well-accepted by users are urgently needed to support current moves toward transdiagnostic, dimensional, and individualized care, and address the significant treatment gap for individuals living with EDs and mental ill-health [171-174]. Importantly, such individuals who are experiencing cognitive challenges as part of their symptom profile are eager for these issues to be addressed in treatment, yet feel these needs are rarely met [35,175,176], experiences that were highlighted in our focus group discussions. Including objective assessment of cognitive impulsivity in a clinical context may thus allow practitioners to identify individuals who would most benefit from targeted cognitive interventions or augmented treatments, and to achieve data-driven personalization of treatment approaches [177-179]. Next steps will therefore involve a detailed psychometric evaluation of the CIS “Papillon Park,” confirming it maintains the properties of the CIS “Choices in the Wild West,” before dissemination. Additional qualitative research is also planned, engaging clinicians and LE participants as key stakeholders to co-design feedback reports and implementation strategies.

The unique contributions of this study include effectively blending user-centered co-design, agile game development, and theory-based cognitive assessment and gamification to reimagine a validated assessment tool. By following up on LE co-design with qualitative and quantitative evaluations, we identified a combination of aesthetics, gamification elements, and accessible playing experiences that maximized user satisfaction, engagement, and motivation while prioritizing psychological safety. This underscores the importance of gamified assessments in the mental health field providing opportunities for users to experience satisfaction of psychological needs, including autonomy, personal achievement, and social support. By effectively engaging users, CIS *Papillon Park* therefore holds promise to facilitate valid and reliable assessment of cognitive impulsivity in individuals experiencing EDs and common co-occurring mental health symptoms. Incorporating well-accepted objective cognitive assessment tools in routine clinical care is needed to support the individualization and prioritization of interventions, and thus support treatment engagement and recovery for these underserved conditions.

## Supplementary material

10.2196/79784 Multimedia Appendix 1 Supplementary materials containing images of the original CIS games, researcher positionality statements, the Game User Experience Satisfaction Scale, short version survey, focus group question guides, and ideas generated during the ideation and design phase.

## Abbreviations

CIS: Cognitive Impulsivity Suite
ED: eating disorder
GUESS-18: Game User Experience Satisfaction Scale, short version
LE: lived experience
NPC: nonplayable character
NSG: Noble Steed Games
REDCap: Research Electronic Data Capture
TA: thematic analysis
