# Supplementary material for: Gamified Assessment of Cognitive Impulsivity in Eating Disorders and Mental Ill-Health: Mixed Methods Study Incorporating Lived Experience Co-Design and Evaluation
Source: JMIR Serious Games. 2026 Jun 3;14:e79784. doi: 10.2196/79784 (PMC13232921; doi:10.2196/79784)
Supplement: Multimedia Appendix 1 [file games-v14-e79784-s001.docx]

**S1. CIS ‘Choices in the Wild West’ games**

1. Bounty Hunter - *Attentional focus* – measuring the ability to direct selective and sustained attention to incoming stimuli, and to initiate appropriate responses and inhibit unhelpful responses to those stimuli.
2. Caravan Spotter - *Information gathering* – measuring tendencies to collect and consider salient information before acting (also referred to as *reflection*).
3. Prospectors Gamble - *Feedback monitoring/shifting* – assessing abilities to flexibly update behaviour in response to positive and negative feedback on previous decisions, and to make beneficial choices under conditions of uncertainty.

**
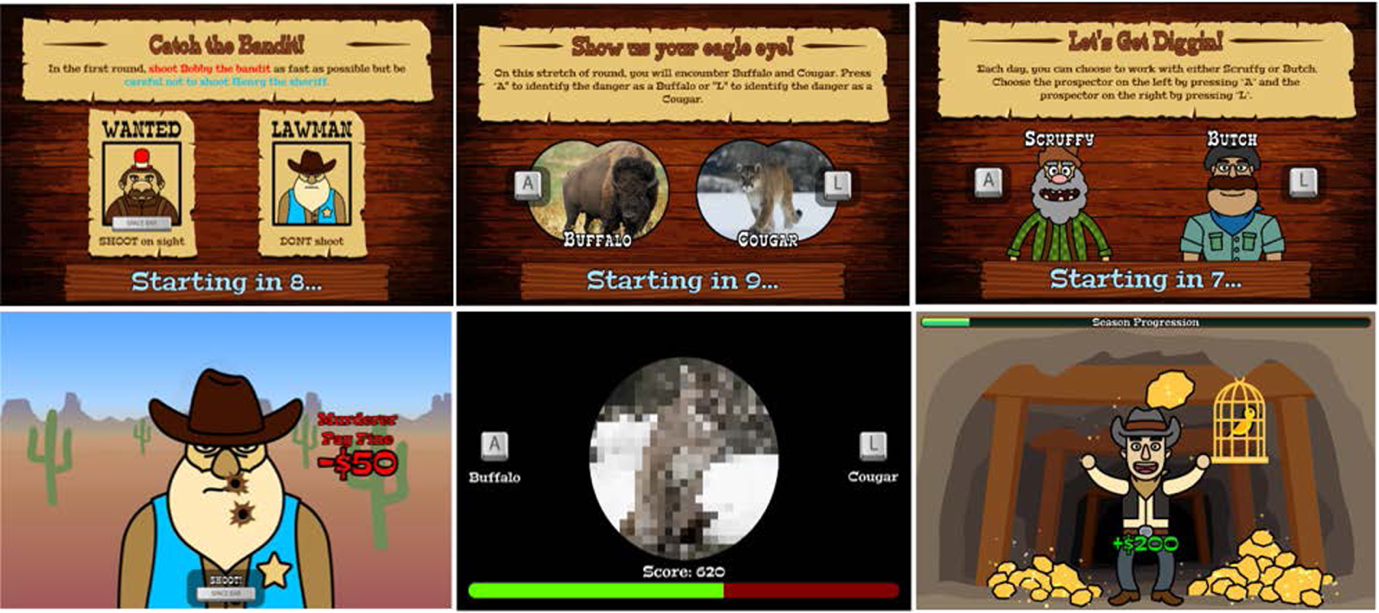
**

Figure S1. Overview CIS ‘Choices in the Wild West’ games.

Reproduced with permission from the author (Verdejo-Garcia et al., 2021).

**S2. Researcher identities and positioning**

The participant-facing members of this research were Emily Colton, Dr Alexandra Anderson, , with support from Dr Lauren Hanegraaf, under the supervision of Professor Antonio Verdejo-Garcia. Researcher identity and positioning statements are reported in Table S2.

Table S2. Researcher roles, identities, and positioning

| **Researcher** |  |
| --- | --- |
| **Emily Colton** | Emily is a cisgender Caucasian woman, aged in her late-40’s. She holds honours degrees in Sociology (University of Leicester, UK) and Psychology (Monash University, Australia).  Emily is currently a PhD candidate, and the primary researcher on this project. As such, her responsibilities included coordinating and co-facilitating all participant sessions, analysing data, acting as client project manager for the game development, and drafting the research report. She approaches her research from a cognitive-behavioural and neuroscience-informed perspective, and values the insights that blending qualitative, quantitative, and lived experience perspectives can provide. |
| **Dr Courtney McLean** | Courtney is a cisgender Caucasian woman, aged in her late-20’s. She holds a PhD in psychology and has experience working with people with eating disorders and mental ill-health, across both qualitative and quantitative research. Courtney was responsible for supporting the qualitative component of this project, including theme development. |
| **Dr Alexandra Anderson** | Alexandra works as a psychologist in an adult trauma focussed clinic. Alexandra previously also worked as a postdoctoral research fellow where she focused on bringing together the voices of researchers, clinicians, and those with lived experiences to improve translational research for eating disorders.  Alexandra is a cisgender Caucasian woman, aged in her late-30s. She has an honours degree in psychology and PhD in clinical psychology.  Alex was responsible for clinical screening of potential participants for this project, as well as co-facilitating focus group sessions. |
| **Dr Lauren Hanegraaf** | Lauren practices as a psychologist in an adult public mental health service and previously worked as a researcher within an eating disorder research team. She values diversity within qualitative research spaces and believes in an inclusive, transdiagnostic approach to mental health research.  Lauren is a cisgender Caucasian woman, aged in her late-20s. She has an honours degree and PhD in psychology. Lauren was positioned alongside the research participants with a focus on supporting psychological wellbeing throughout the research process. |

**S3. Materials**

**S3.1 Game Evaluation Questionnaire**

Game User Experience Satisfaction Scale (GUESS-18) (Keebler et al., 2020; Phan et al., 2016).

Please think of the suite of CIS cognitive assessment games that you recently played.

Based on your experience of playing this game, please rate the following statements on a seven-point scale ranging from "Strongly Disagree" to "Strongly Agree".

| **Subscale** | **Item** | **Item #** |
| --- | --- | --- |
| Usability/Playability | I find the controls of the game to be straightforward. | 1 |
|  | I find the game’s interface to be easy to navigate. | 11 |
| Narratives | I am captivated by the game’s story from the beginning. | 2 |
|  | I enjoy the story provided by the game | 12 |
| Play Engrossment | I feel detached from the outside world while playing the game. | 3 |
|  | I do not care to check events that are happening in the real world during the game | 13 |
| Enjoyment | I think the game is fun | 5 |
|  | I feel bored while playing the game [reverse coded] | 6 |
| Creative Freedom | I feel the game allows me to be imaginative. | 4 |
|  | I feel creative while playing the game | 14 |
| Audio Aesthetics | I enjoy the sound effects in the game | 7 |
|  | I feel the game’s audio (e.g., sound effects, music) enhances my gaming experience | 15 |
| Personal Gratification | I am very focused on my own performance while playing the game. | 8 |
|  | I want to do as well as possible during the game | 16 |
| Social Connectivity | I find the game supports social interaction | 9 |
|  | I enjoy the social interactions within the game. | 17 |
| Visual Aesthetics | I enjoy the game’s graphics | 10 |
|  | I think the game is visually appealing. | 18 |

Based on your experience of playing the CIS cognitive assessment games, please continue to rate the following statements on a seven-point scale ranging from "Strongly Disagree" to "Strongly Agree".

| No Trigger Warnings Needed | I feel the games are triggering for someone with lived experiences like mine [reverse coded] | NA |
| --- | --- | --- |
|  | I feel the games are appropriate for someone with lived experiences like mine | NA |

**Table S3.2 Game evaluation questionnaire internal consistency**

| **Subscale** | **Alpha**  **(Cronbach, 1951)** | **Ordinal Alpha**  **(Zumbo et al., 2007)** |
| --- | --- | --- |
| **GUESS-18 Total** | **0.930** | **0.947** |
| Usability/Playability | 0.877 | 0.999 |
| Narratives | 0.827 | 0.834 |
| Play Engrossment | 0.009 | 0.150 |
| Enjoyment | 0.695 | 0.750 |
| Creative Freedom | 0.916 | 0.959 |
| Audio Aesthetics | 0.921 | 0.944 |
| Personal Gratification | 0.682 | 0.719 |
| Social Connectivity | 0.613 | 0.816 |
| Visual Aesthetics | 0.816 | 0.889 |
| Internal consistency is interpreted as >0.90 = excellent, 0.80-0.89 = good, 0.70-0.70 = acceptable, 0.60-0.69 = questionable, 0.50-0.59 = poor, 0.00-0.49 = unacceptable. | | |

**Table 3.3 Alignment of Themes with GUESS-18 Items**

| **Theme** | **Subscale** | **Item** |
| --- | --- | --- |
| NA | Usability/Playability | I find the controls of the game to be straightforward. |
|  |  | I find the game’s interface to be easy to navigate. |
| Aesthetic appeal and a progressive story create an immersive user experience | Narratives | I am captivated by the game’s story from the beginning. |
|  |  | I enjoy the story provided by the game |
|  | Visual Aesthetics | I enjoy the game’s graphics |
|  |  | I think the game is visually appealing. |
|  | Audio Aesthetics | I enjoy the sound effects in the game |
|  |  | I feel the game’s audio (e.g., sound effects, music) enhances my gaming experience |
| Achieving individual and collaborative goals fosters player motivation and purpose | Personal Gratification | I am very focused on my own performance while playing the game. |
|  |  | I want to do as well as possible during the game |
| Relatedness, variety, and autonomy: fulfilling key psychological needs enhances user engagement | Social Connectivity | I find the game supports social interaction |
|  |  | I enjoy the social interactions within the game. |
|  | Creative Freedom | I feel the game allows me to be imaginative. |
|  |  | I feel creative while playing the game |
| NA | Play Engrossment | I feel detached from the outside world while playing the game. |
|  |  | I do not care to check events that are happening in the real world during the game |
| NA | Enjoyment | I think the game is fun |
|  |  | I feel bored while playing the game [reverse coded] |

**Table S3.4 Additional evaluation questionnaire results**

|  | **All**  **(*N* = 18)** | **Designers**  **(*N* = 5)** | **Zoom**  **(*N* = 6)** | **Own Time**  **(*N* = 7)** |
| --- | --- | --- | --- | --- |
| *No Trigger Warnings Needed* | 5.72 (1.35) | 6.7 (0.45) | 6.08 (0.58) | 4.71 (1.29) |
| Note: Ratings differed significantly between research groups, *F*(2,15)=7.64, *p<*.01. Tukey’s HSD post-hoc tests showed the ratings in the *Own Time* group were significantly lower than the *Designers*, *p*<.01 and the *Zoom* group, *p*<.05. | | | | |

**S4. Design phase documentation**

**S4.1 Focus Group 1, Question Guides and Participant Briefings**

Our aim for today is to come up with ideas to redesign a set of three cognitive assessment games. The three games should fit together within a common theme or context, such that each contributes toward an overall goal, purpose, or mission. To redesign the three cognitive assessment games, we would like your help to choose:

- The overall theme / context
- Goals and objectives for the games
- Characters
- Style, Colour palate, etc

We will first talk about this at a general level, then break for morning tea, and then dig into the specifics for each of the three games.

Q1. Let’s start by thinking about some of the themes or contexts that have appeared in computer games you have played or seen before.

Probes:

- What is fun and engaging about these themes?
- What do they look like (colours, scenes, etc)?
- What do you like / dislike about these themes and the way they look?
- Do they appeal more to some groups of people than others? Why?
- Is there anything about these themes you find helpful or harmful in relation to your lived experiences? Why/how?

Q2. With these fun themes in mind, what are some ideas for an overall game purpose or goal?

Probes:

- What individual tasks would someone need to perform to meet that goal?
- What is fun and engaging about these goals / tasks?
- How will someone know if they are being successful (how are they rewarded)?
- What tools or resources might help accomplish these goals/tasks?
- What items or features might get in the way?

Q3. What characters might exist within these themes or contexts?

Probes:

- Who might the main character be?
- What are some character features that might appeal to the broadest possible audience?
- What other characters might they encounter?
- What types of characters might be friends or enemies?
- How might other characters help or hinder the player in accomplishing their goals?

Q4. Before we break for Morning Tea, what do we need to be careful or sensitive about?

Probes:

- What might be upsetting or offensive?
- What might be frustrating?
- Is there anything we have discussed that could be triggering for someone with lived experiences like yours?

30 Minute Break for Morning Tea – we will reconvene to dig into the specific of the three games.


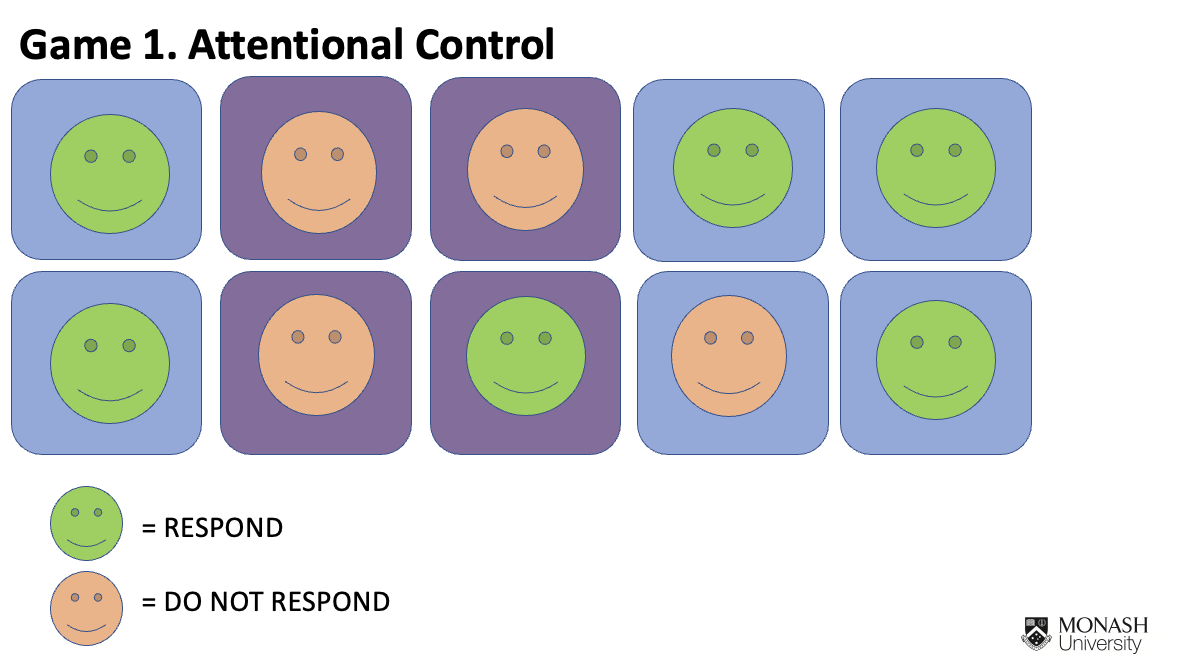


- Players are presented with one of two visual stimuli. The goal is to respond quickly to one but to withhold a response to the other within a limited time (1 second).
- Stimuli are presented in two different backgrounds or environments. Most often, one background occurs when the response is required, and the other background occurs when no response is required, therefore helping the player select the correct responses. However, sometimes they swap, so they challenge accurate responding. The time between the background appearing and the stimulus appearing varies.
- Goals are speed and accuracy. Points are awarded for accurate responses and lost for inaccurate responses non-responses.
- There are four blocks of this game.

Q5. Having seen the basics of this game’s structure, what task could xx character perform in xxx context that fit with this game structure?

Probes:

- What is the overall theme / context / task?
- A game goal (i.e., what should players ‘respond’ to or ‘not respond’ to? Why are they ‘responding’ or ‘not responding’?)
- Two backgrounds / environments (should be relatively similar)
- Four pairs of stimuli, with each pair being relatively similar in size, shape, and colours (we need four pairs because there are four game blocks, and the stimuli change in each block).


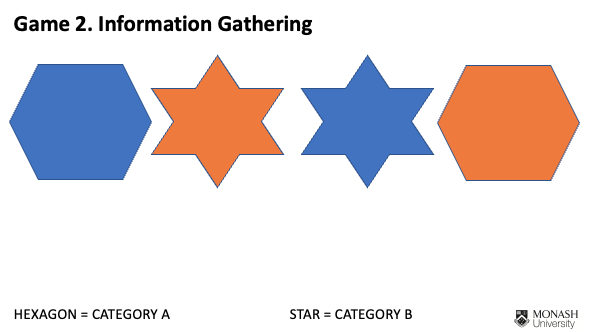


- Players are presented with an ambiguous, pixelated image, which gradually ‘de-pixelates’ (becomes clearer with time – slide animations represent this) into an example from one of two categories of stimuli.
- Players need to watch long enough to decide whether the image is from category A or B as it is de-pixelating. However, their goal is speed as well as accuracy.
- Points are awarded for accurate responses and lost for inaccurate responses and higher points are awarded for faster correct responses.
- There are four blocks of this game.

Q6. Having seen the basics of this game’s structure, what task could xx character perform in xxx context that fit with this game structure?

Probes:

- What is the overall theme / context / task?
- What is the goal (i.e., why would the player have to quickly work out which category the pixelated image reflects?)
- What are some similar pairs of categories (they need to be similar because it should be difficult to work out which category the image is when it is very pixelated/ambiguous).


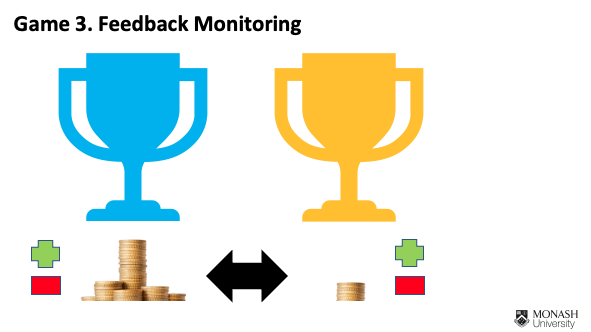


- Players are repeatedly presented with two stimuli, with the goal to choose the ‘best’ alternative each time.
- Each stimulus is associated with different probabilities of points outcomes – these may be a large win, small win, neutral, small loss, or large loss.
- Probabilities and outcomes remain stable for a time and then change.
- The overall goal is to accumulate the most possible points.
- There are five blocks of this game.

Q7. With the basics of this final game’s structure in mind, what task could xx character perform in xxx context that fit with this game structure?

Probes:

- What is the overall theme / context / task?
- What is the goal (i.e., why/how would the player be rewarded for choosing one stimulus and not the other? What is the reward?)
- What are some ideas for a pair of stimuli? (with no obvious qualitative differences)

**S4.2 Focus group experience rating scale**

**Focus Group Session 1 Results**

|  | **Q1** | **Q2** | **Q3** | **Q4** | **Q5** |
| --- | --- | --- | --- | --- | --- |
| Participant 1 | 9 | 9 | 9 | 10 | 10 |
| Participant 2 | 10 | 10 | 10 | 5 | 10 |
| Participant 3 | 10 | 10 | 10 | 10 | 10 |
| Participant 4 | 10 | 8 | 8 | 10 | 9 |
| Participant 5 | 10 | 10 | 7 | 10 | 10 |
| Participant 6 | NA | NA | NA | NA | NA |

Note: Higher scores indicate more positive experiences.

Free-text feedback: “Friendly environment, great variety of contributors, and a distinct lack of perceived pressure surrounding decisions. Overall, a great experience. Thank you.”

**Focus Group Session 2 Results**

|  | **Q1** | **Q2** | **Q3** | **Q4** | **Q5** |
| --- | --- | --- | --- | --- | --- |
| Participant 1 | NA | NA | NA | NA | NA |
| Participant 2 | 10 | 10 | 10 | 10 | 10 |
| Participant 3 | NA | NA | NA | NA | NA |
| Participant 4 | 10 | 10 | 10 | 10 | 10 |
| Participant 5 | 10 | 9 | 10 | 10 | 10 |
| Participant 6 | NA | NA | NA | NA | NA |

Note: Higher scores indicate more positive experiences.

Free-text feedback: “I think the session was run respectfully and inclusively. We were all given a chance to speak, and I felt like nobody's voices were considered less important or given less airtime. I enjoyed the session - adequate number and duration of breaks, food was great.” **S4.3 Focus Group 2 Question Guide**

RECAP:

We are aiming to re-design a game called the Cognitive Impulsivity Suite, which tests some important thinking processes and behavioural tendencies that people experience:

- Little reflection on information.
- Difficulty controlling our attention / inhibiting responses.
- Difficulty choosing between uncertain options.

**Why**? We want to make it more appealing for a broader audience.

The CIS 2.0 may help us to better understand cognitive processes involved in eating disorders and other mental health difficulties and may inform the development of novel treatments.

1. **Attentional Control**

Q1. How do you feel about:

- - The overall theme and task
  - The game goals (i.e., to what and why players are ‘responding’ or ‘not responding’?)
  - The backgrounds / environments
  - The characters / stimuli

**2. Information Gathering**

Q2. How do you feel about:

- - The overall theme and task
  - The game goals (i.e., why the player has to quickly work out which category the image represents)
  - The categories / stimuli.

**3. Feedback Monitoring / Shifting**

Q3. How do you feel about:

- - The overall theme and task
  - The game goals (i.e., why/how the player is rewarded for choosing one stimulus and not the other; the reward they receive/lose)
  - The characters / stimuli.

Q4. Overall, how do you feel about:

- - The overall theme
  - The characters
  - The opportunities for character personalisation
  - The colours / style

**S4.4 Focus Group 1 Summary of Ideas**

1. **Attentional Control**

| **Backgrounds (Pairs)** | | |
| --- | --- | --- |
| Geographical locations | Global landmarks | Coliseum, Eiffel Tower, Buckingham Palace, Giza Pyramids, Aztec temple |
|  | Natural environments | Forest, Lake, Mountain; city vs country |
|  | Buildings or rooms | School, shop, inn |
| Contrasting conditions | Seasons | Shown by conditions of trees in the background |
|  | Weather | Sunny or raining; dramatic conditions such as storms, tornadoes |
|  | Lighting | Night vs Day, traffic lights, indoor lighting on/off |
|  | Doors / Shutters / Traps | Open vs closed; present or absent; one type or another |
| Degree of density | Town square | Crowded vs few people |
|  | Animals | Herd in a field; pets in a store; fish in a pond or lake |
|  | Tree / Shrub | Fully laden with flowers or fruit vs bare or sparse |
|  | Barrel / Container | Full vs empty; or containing something specifics vs a contrasting item |
|  | Traffic | Busy vs quiet traffic conditions; particular types of vehicles |
| **Stimuli (Pairs)** | | |
| Congruency with backgrounds | Uniform or Disguise | ‘Correct’ items that will allow the player to blend in or pass as a member of a group – e.g., Roman soldier, palace guard – vs an alternative that will stand out / does not fit with the background |
|  | Clothing | Appropriate to the context and task – e.g., climbing shoes for a mountain, warm hat for winter |
|  | Characters | Friendly locals; a particular person you’re searching for |
|  | Animals | Yours or in another way the ‘right’ one vs the wrong one |
|  | Keys | Matched to a door or chest etc in the background |
| Helpful vs Hopeless | Tools / Resources | e.g. umbrella vs parasol (useful in the weather conditions); a modern or effective tool vs an old or broken counterpart |
|  | Transport | Get you further on your journey or able to carry more with you – e.g., bike vs motorbike; bus |
|  | Real / Fake versions | E.g., horse vs rocking horse |

1. **Reflection**

Many of the ideas above can also work in this game, in particular the helpful vs hopeless and congruent vs incongruent stimulus pairs. Additional ideas for goals, pixelation, and stimuli are listed below. Generally, the participants wanted four different versions rather than the same one four times over, and for it to fit with the environment / stage of the journey / context of the story at that moment.

| **Goals** | | |
| --- | --- | --- |
| Necessary for progress in the journey / story | Transport | Fast vs slow versions (e.g., bike vs motorbike; racehorse vs old donkey); large & spacious vs small & limited capacity |
|  | Equipment / Resources | Dressing for the weather; modern & effective vs old or broken; large vs limited capacity; retrieving a lost or dropped item before it disappears or breaks |
|  | Companions / Teammates / Community Members | Finding the right person; rescuing people; |
|  | Animals | Useful vs useless alternatives; yours vs not; odd-one-out |
|  | Acquiring skill / experience / knowledge | Clues to solve mysteries; ‘leveling up’ the playing character by learning or practicing the skill or ability that will achieve the game goal – speed gains more |
|  | Acquiring the game ‘currency’ | Whatever the ‘thing’ is that will achieve the ultimate game goal – a potion to return the player or community to its original state; a fertilizer to grow the biggest and best garden – speed acquires more |
| **Pixelation** | | |
|  | Water | Stimuli (items; people; fish) bobbing up from beneath the surface; bubbles slowly clearing |
|  | Containers | Chest or shell slowly opening to reveal the target or low-value alternative |
|  | Weather conditions | Clouds gradually parting; Rain or fog gradually dissipating |
|  | Crowds | Gradually thinning or stopping moving; target comes closer or becomes easier to identify |
|  | Dust | Storm or cloud gradually dissipates |
|  | Clutter | Gradually thinning or target comes closer or becomes easier to identify |
|  | Animals | Herd gradually thinning or spreading out; fur flying |
|  | Focus | Binoculars or glasses gradually bringing stimuli into focus |
|  | Light | Beam of light (e.g., flashlight) gradually growing wider; light getting brighter |
|  | Magic | Magic spell gradually dissipating; |
| **Stimuli (Pairs)** | | |
|  | Fast vs Slow | Animals or vehicles or fuels that accelerate or slow down your journey |
|  | Helpful vs Hopeless | Types of umbrella / clothing / maps / keys / other tools that are appropriate vs inappropriate to the context & environment |
|  | Treasure vs Trash | Pearl vs bottle caps; treasure vs buttons; high potency potion or medicine or fuel vs ineffective alternative |
|  | Yours vs Not | Companion / community member / teammate; pet or animal companion; lost or dropped items |
|  | Real vs Fake | As above, e.g., a real animal vs a toy version; sword vs balloon sword |
|  |  |  |

1. **Feedback Monitoring / Shifting**

As above, generally, the set of choice alternatives and resulting rewards and punishments should be consistent with the overall story and the game environment or context at the given time.

| **Choice Alternatives (Pairs)** | | |
| --- | --- | --- |
| Characters | Guides | Lead you to a reward or make you lose what you already have; lead you to a destination quickly or get you lost |
|  | Craftspeople / Makers | Build or create you useful or broken versions (e.g., boatbuilders constructing boats and oars, chemists mixing up potions or fuels; pharmacist creating medicines) |
|  | Teachers / Experts | Provide knowledge or help the player acquire a skill, or hinder them doing so |
|  | Officials | Provide help or access, or hinder your progress – e.g., guard, police officer, government official |
|  | Other community members | Provide help or create mischief (e.g., shopkeeper, innkeeper, hairdresser, florist); have attributes that are a good fit for the environment or not |
|  | Animals | Helpful or hinder – e.g., a dog that leads you to a rewards vs one that runs away |
| Items | Transport | Vehicles that are either faster or slower; larger allowing you to carry more goods or people, or smaller; effective or break down; make progress or go backwards |
|  | Gear / Equipment | Tools that are modern and effective in building or acquiring something, or old and broken or cause something to be lost or broken |
|  | Containers | Hold the reward / currency or something that causes the player to lose some they have already acquired |
|  | Clothing | Allow you to progress in the journey or acquire other rewards, or cause you to fall behind or lose what you’ve already acquired |
| Environments | Plants / Gardens / Forests | Grow more flowers or fruit, or product few or ones that are rotten or provoke allergic reactions |
|  | Paths or trails | Fast/easy routes to a particular destination or a reward, or those that are slow/difficult or lead the wrong way or to a punishment |
|  | Geographical locations | ‘Choose your own adventure’ element – go to the city or country; to the forest or the mountains – in each place you find or lose what you need to achieve the ultimate game goal |
| Other | Magic | Wands to perform spells; cauldrons, or ingredients to mix potions; |
| **Rewards / Punishments** | | |
|  | Game ‘currency’ | Gold, treasure, or other valuable tokens that can be traded; potion or medicine that returns the player or community to their original state; fuel that powers a vehicle for the journey |
|  | Items & resources | Finding or losing; collecting and carrying vs dropping or spilling them’ being given or having them taken away or stolen |
|  | Speed / progress | Assisted or hindered in the journey or mission |
|  | Experience & skills | Learning, practicing, and mastering vs failing or getting worse |
|  | People or animals | Added to or lost from your team or community |
|  |  |  |

**S4.5 Focus Group 2 – Summary of Ideas**

| **General** | | |
| --- | --- | --- |
|  | Context | The player is a trainee Mage, traveling alongside an experienced mentor to acquire the skills and experience they need for the ultimate game goal  (alternative gender-neutral terms for magical characters include Magician, Sorcerer, Mystic, Seer, Enchanter, Spellcaster, etc). |
|  | Characters | Trainee (not depicted in the game);  Mentor; (depicted in the game with limited options for customisation);  Animal side-kicks of the trainee and mentor (cat, frog, newt, owl, etc – also may have options for customisation);  Members of the broader community (villagers);  Magical or mythical creatures (e.g., dragon, phoenix, unicorn, griffin). |
|  | Goal / purpose | Learning, practicing, and mastering magical skills – potion making, spell-casting, transmutation, divination, telekinesis, healing, animal communication, etc. |
|  | Game ‘currency’ | Potion - performance in each game is demonstrated by accumulated more potion, with a visual (bar filling with purple colour; bottle and vials accumulating on a shelf) provided to the player. |
|  | Prosocial Purpose | The potion will be used to: return members of the community to their original state; fuel the travelling wagon; proves the trainee’s skill to be qualified and/or hired by the mentor; provide fertilizer to grow the biggest and best garden; create a habitat or food for magical creatures. |
|  | Values / Sensitivities | Being careful regarding gender representation, body image, food and eating, trauma and violence; the punishments and the ultimate game goal are gentle and not dangerous, menacing, or harmful. |
|  | Variety & personalisation | Ability to customise some features such as clothing (hats, cloaks, etc); the side-kick (type or colour of the animal);  As much variety as can be built in without compromising the scientific structure – e.g., backgrounds become distracting, difficulty keeping track of the cue or prompt, etc. |
|  | Broad Appeal | Appealing, ‘friendly’ characters; bright colours; child-like and playful; |
|  |  |  |

| **Attentional Control** | | |
| --- | --- | --- |
| Environments | Natural environments | Forest, Lake, Swamp, Mountain, Plains, Desert – indicate progression of the journey and provide variety |
|  | Travelling Wagon | Inside the wagon – different environments visible through an open door or window |
| Background cues - contrasting conditions (Pairs) | Seasons | Shown by conditions of trees and plants in the background |
|  | Weather | Sunny & clear or cloudy & raining |
|  | Lighting | Night vs Day; indoor lighting on/off |
|  | Doors / Shutters | Open vs closed; change of outside environment |
| Stimuli (Pairs) - Correct vs incorrect ingredient – the ‘correct’ items will allow the player to produce more potion | Plants – e.g., that would be found in a forest; | Congruent with the story and position along the journey; appropriate or mismatched to the natural environment and cues; differ according to something other than colour (colour-blindness sensitivity). |
|  | Natural items – e.g., a feather from the kind of bird that would be found in that environment; sand / snow / rocks / water |  |

Note: Frog and ingredient need to be larger and front/centre (Mage off-centre and further back; cauldron further back).

| **Information Gathering** | | |
| --- | --- | --- |
| Goals & rewards | Acquiring the in-game ‘currency’ | Accumulating potion that will achieve the ultimate game goal – speed & accuracy acquires more |
|  | Speed of response (why is this necessary?) | Simply to gain more potion; to acquire the target before it decays or changes or is lost; another imperative that fits with the story and the background / environment for each block |
| Disambiguation | Magical Spell | Cloud or sparkles gradually dissipate revealing the result of the spell casting. |
|  | Egg | Gradually cracks or splits open to reveal a hatchling newt or dragon (or similar) |
|  | Rock | Has been transmuted – gradually transforming back into stimuli (for ideas, see below). |
| Target Stimuli (Pairs) | Animals (e.g., identify which of two types while a baby before they grow and develop; grab your side-kick instead of an imposter or before they disappear or a spell takes effect) | Useful / desirable or neutral / undesirable (but not dangerous or harmful); matched to the game context and environment; ideally a different pair and matched background for each block |
|  | Plants (e.g., grab before they wilt) |  |
|  | Magical tools / resources (e.g., grab a crystal ball instead of a useless shiny rock or a tennis ball; grab a wand instead of a stick or pencil; a carriage before it turns back into a pumpkin; a horse before it turns back into a mouse) |  |
|  | Treasure (e.g., grab the gold before it becomes lead or the diamond before it turns back into coal; |  |
|  | People (e.g., identify a particular NPC you need to talk to or rescue or learn from vs a mischievous alternative; |  |

| **Feedback Monitoring / Shifting** | | |
| --- | --- | --- |
| **Choice Alternatives** (Pair of similar stimuli – no obvious qualitative differences) | Goblin Potion-Bottlers | Pick which goblin is allowed to bottle the potion – goals and rewards |
|  | Another magical creature or expert or tool | Pick which (wizard, pixie, etc) can best teach you another magical skill; pick which crystal ball will help you find the most magical plants / eggs / potion, etc. |
| Rewards & Punishments | Potion as the consistent in-game currency | Bottles of potion in same proportions as the gold / money gained or lost in the original game ($0/$50/$200 translates to 0/1/4 bottles or a multiple thereof) |

S6. **Supplementary References**

Cronbach, L. J. (1951). Coefficient alpha and the internal structure of tests. *Psychometrika*, *16*(3), 297-334. <https://doi.org/10.1007/BF02310555>

Keebler, J. R., Shelstad, W. J., Smith, D. C., Chaparro, B. S., & Phan, M. H. (2020). Validation of the GUESS-18: a short version of the Game User Experience Satisfaction Scale (GUESS). *Journal of Usability Studies*, *16*(1), 49. <https://commons.erau.edu/publication/1508?utm_source=commons.erau.edu%2Fpublication%2F1508&utm_medium=PDF&utm_campaign=PDFCoverPages>

Phan, M. H., Keebler, J. R., & Chaparro, B. S. (2016). The development and validation of the Game User Experience Satisfaction Scale (GUESS). *Human Factors*, *58*(8), 1217-1247. <https://doi.org/10.1177/0018720816669646>

Verdejo-Garcia, A., Tiego, J., Kakoschke, N., Moskovsky, N., Voigt, K., Anderson, A., Koutoulogenis, J., Lubman, D. I., & Bellgrove, M. A. (2021). A unified online test battery for cognitive impulsivity reveals relationships with real-world impulsive behaviours. *Nature Human Behaviour*, *5*(11), 1562-1577. <https://doi.org/10.1038/s41562-021-01127-3>

Zumbo, B. D., Gadermann, A. M., & Zeisser, C. (2007). Ordinal versions of coefficients alpha and theta for Likert rating scales. *Journal of Modern Applied Statistical Methods*, *6*(1). <https://doi.org/10.22237/jmasm/1177992180>
